# Anticoronavirus Isoquinoline Alkaloids: Unraveling the Secrets of Their Structure–Activity Relationship

**DOI:** 10.1111/irv.70166

**Published:** 2025-10-05

**Authors:** Marcela Safratova, Yu‐Li Chen, Anna Hostalkova, Jakub Chlebek, Chung‐Fan Hsieh, Bing‐Hung Chen, Lucie Cahlikova, Stefan Kosturko, Anders Backlund, Jim‐Tong Horng, Tsong‐Long Hwang, Michal Korinek

**Affiliations:** ^1^ Department of Pharmacognosy and Pharmaceutical Botany Faculty of Pharmacy, Charles University Hradec Kralove Czech Republic; ^2^ Center for Drug Research and Development, College of Human Ecology Chang Gung University of Science and Technology Taoyuan Taiwan; ^3^ Graduate Institute of Health Industry Technology, College of Human Ecology Chang Gung University of Science and Technology Taoyuan Taiwan; ^4^ Research Center for Emerging Viral Infections, College of Medicine Chang Gung University Taoyuan Taiwan; ^5^ Molecular Infectious Disease Research Center, Chang Gung Memorial Hospital Chang Gung University College of Medicine Taoyuan Taiwan; ^6^ Department of Biotechnology, College of Life Science Kaohsiung Medical University Kaohsiung Taiwan; ^7^ Department of Pharmaceutical Biosciences, Faculty of Pharmacy Uppsala University Uppsala Sweden; ^8^ Department of Biochemistry and Molecular Biology, College of Medicine Chang Gung University Taoyuan Taiwan; ^9^ Graduate Institute of Natural Products, College of Medicine Chang Gung University Taoyuan Taiwan; ^10^ Department of Anesthesiology Chang Gung Memorial Hospital Taoyuan Taiwan; ^11^ Graduate Institute of Natural Products, College of Pharmacy Kaohsiung Medical University Kaohsiung Taiwan; ^12^ Department of Medical Research, Kaohsiung Medical University Hospital Kaohsiung Medical University Kaohsiung Taiwan; ^13^ Drug Development and Value Creation Research Center Kaohsiung Medical University Kaohsiung Taiwan

**Keywords:** bis‐benzylisoquinoline, ChemGPS‐NP, docking, Omicron, SARS‐CoV‐2

## Abstract

**Background:**

Natural alkaloids are a structurally diverse class of bioactive compounds with significant therapeutic potential. This study aimed to evaluate the in vitro antiviral activity of various natural alkaloids against coronaviruses, clarify molecular effects via bioassays and docking, and explore structure–activity relationships. Tested compounds included a wide variety of isoquinoline and Amaryllidaceae‐type alkaloids.

**Methodology:**

Antiviral activity was assessed using HCoV‐229E and pseudotyped lentivirus assays for different strains of severe acute respiratory syndrome coronavirus 2 (SARS‐CoV‐2). Cytotoxicity was evaluated with the WST‐1 assay. AutoDock was used for molecular docking, online tools assessed drug‐likeness, and ChemGPS‐NP analyzed physicochemical properties correlated to antiviral clinical drugs.

**Results:**

Several bis‐benzylisoquinoline alkaloids, especially from *Berberis vulgaris* L., and specific Amaryllidaceae alkaloids showed protective activity against HCoV‐229E (EC_50_ = 4.1–8.1 μM). Active compounds were further tested against SARS‐CoV‐2 variants. Aromoline (Compound **16**) exhibited strong antiviral activity, inhibiting D614G, Delta, and Omicron variants in pseudovirus assays with IC_50_ values of 0.47–0.66 μM. Other bis‐benzylisoquinoline analogues showed moderate activity (IC_50_ = 1.24–2.86 μM). Docking studies revealed aromoline's favorable interaction at the SARS‐CoV‐2 spike/ACE2 interface, forming hydrogen bonds with Gln493 and Ser494 (binding energy −5.34 kcal/mol). ChemGPS‐NP analysis highlighted a distinct cluster of active bis‐benzylisoquinolines (Compounds **16**–**19**) in chemical space.

**Conclusion:**

This study highlights the antiviral potential of bis‐benzylisoquinoline and Amaryllidaceae alkaloids, particularly aromoline. The findings support their relevance as scaffolds for developing novel anticoronavirus agents and advance the understanding of their structure–activity relationships.

**Abbreviations**
3CLpro: 3C‐like protease
AAs: Amaryllidaceae alkaloids
ACE2: angiotensin‐converting enzyme 2
ACE2‐293T: ACE2 expressing cells
BBI: bis‐benzylisoquinoline alkaloids
COVID‐19: coronavirus disease 2019 caused by SARS‐CoV‐2 infection
hCoV: human coronavirus
Huh7: human liver carcinoma cell line
IAs: isoquinoline alkaloids
Mpro: SARS‐CoV‐2 virus main protease
RBD: receptor‐binding domain
SAR: structure–activity relationship
SARS‐CoV‐2: severe acute respiratory syndrome coronavirus 2
S‐protein: spike protein
TMPRSS2: transmembrane serine protease 2

## 1 Introduction

Coronaviruses are single‐stranded viruses that affect mammals and birds. The coronavirus epidemic attracted attention in 2003 with the SARS outbreak; then, in 2019, severe acute respiratory syndrome coronavirus 2 (SARS‐CoV‐2) was identified as the seventh known human‐infecting coronavirus. This virus, from the genus *Betacoronavirus*, Coronaviridae family, is an enveloped single‐stranded RNA virus that usually enters the body through the respiratory tract. The envelope is a lipid membrane surrounding its protein capsid. SARS‐CoV‐2 enters host cells by binding spike protein (S‐protein) on the viral particle membrane and angiotensin‐converting enzyme 2 (ACE2) receptors located on the surface of host cells [1]. Consequently, the virus infects human organs where ACE2s are widely distributed, including the lungs, heart, and kidneys. SARS‐CoV‐2, which causes COVID‐19, has spread globally, infecting around 778 million people and causing over 7 million deaths, significantly disrupting global education, business, and the economy due to lockdowns [2].

SARS‐CoV‐2 has several target proteins, including nonstructural proteins Mpro (the main protease), also known as 3CLpro (3C‐like protease), the PLpro (papain‐like “cysteine” protease), the RNA‐dependent RNA polymerase, and the structural glycoprotein embedded on the virus envelope, S‐protein (spike protein) [3]. The S‐proteins interact directly with human ACE2, essential for the virus's entry into the cells [1]. Computational methods have been applied to screen for drugs against human coronavirus (HCoV) [3, 4]. For example, several alkaloidal drugs have been identified with potential against 3CLpro, which controls virus replication [5].

Seven types of the coronavirus family are known to infect humans, binding to receptors via S‐proteins during the early stages of their life cycle. Although HCoV S‐proteins are similar, the structural difference leads to receptor specificity [6]. For example, HCoV‐229E binds to aminopeptidase N; MERS‐CoV to dipeptidyl peptidase‐4; HCoV‐NL63, SARS‐CoV, and SARS‐CoV‐2 to ACE2 [7]; and the β1‐coronaviruses (including HCoV‐OC43) and HCoV‐HKU1 employ glycan‐based receptors carrying 9‐*o*‐acetylated sialic acid [8]. Recent SARS‐CoV‐2 mutations, such as Omicron, are causing severe acute respiratory distress syndrome with significant inflammatory responses [9].

Natural products, particularly alkaloids, are promising sources of antiviral agents. With nearly 50% of small‐molecule drugs derived from natural products, the search for new antiviral compounds has intensified [10]. Alkaloids, known for their broad‐spectrum antiviral properties, have shown potential against neurodegenerative diseases and viruses like Zika, H5N1, and HSV1, with recent research on Amaryllidaceae alkaloids (AAs) and isoquinoline alkaloids (IAs) highlighting their cytotoxic and antiviral activities [11, 12]. Most studies on alkaloid anticoronavirus effects have been conducted in silico, making our study's in vitro evaluation of different alkaloid structures using established anticoronavirus models valuable [13, 14, 15]. To address this gap, we employed a well‐established HCoV‐229E infection assay using Huh7 cancer cells and a lentivirus‐based SARS‐CoV‐2 entry assay utilizing luciferase as a reporter to quantify viral entry efficiency in hACE2‐overexpressing HEK293T cells [16]. To better understand the molecular basis of antiviral activity, a structure–activity relationship (SAR) analysis was performed to correlate specific structural features of the active alkaloids with their inhibitory effects against SARS‐CoV‐2.

The current study aimed to evaluate the in vitro antiviral activity of various natural isoquinoline and Amaryllidaceae‐type alkaloids against coronaviruses, investigate their molecular effects through bioassays and docking studies, and explore SARs.

## 2 Materials and Methods

**AAs**: (±)‐Haemanthidine (**1**), (+)‐hamayne (**2**), (+)‐haemanthamine (**3**), 9‐*O*‐demethylgalanthine (**4**); lycorenine (**7**), oduline (**8**), seco‐isopowellaminone (**6**); lycorine (**9**); homolycorine (**10**), masonine (**11**), hippeastrine (**12**); (+)‐tazettine (**5**); (−)‐undulatine (**13**), (−)‐crinine (**14**), (−)‐caranine (**15**). **IAs**: Allocryptopine (**29**), (+)‐bulbocapnine (**28**), (+)‐canadaline (**30**), (+)‐canadine (**26**), (−)‐corypalmine (**25**), (+)‐corydaline (**31**), and (+)‐thalictricavine (**27**); tetrahydrocolumbamine (**32**); argemonine (**23**), (−)‐caryachine (**34**), (−)‐*O*‐methylcaryachine (**35**), *O*‐methylneocarychine (**36**), (−)‐scoulerine (**33**); 6‐ethoxydihydrosanguinarine (**51**), 6‐ethoxydihydrochelerytrine (**52**), chelidonine (**50**), (−)‐stylopine (**49**); papaverine (**53**), thebaine (**54**), (−)‐narcotine (**55**); *N‐*methyllaurotetanine (**56**), (−)‐salutaridine (**59**), (−)‐pallidine (**60**), norisocoridine (**57**), (+)‐laurotetanine (**58**); 8‐oxoberberine (**22**), (+)‐aromoline (**16**), (+)‐obamegine (**19**), (+)‐berbamine (**17**), (+)‐bersavine (**18**), (−)‐muraricine (**20**), (−)‐berkristine (**21**); (−)‐fumarophycine hydrochloride (**37**), (−)‐fumaricine (**38**), (+)‐parfumine (**42**), (−)‐sinactine (**43**), (+)‐fumariline (**41**), (±)‐*O*‐methylfumarofine (**44**), cryptopine (**45**), (+)‐bicuculline (**46**), (−)‐*O*‐methylfumarophycine (**39**), (−)‐fumaritine (**40**); norchelidonine (**48**); (−)‐platycerine (**24**); glaucine (**47**), oxoglaucine (**61**), and liriodenine (**62**) (Scheme S1). For detailed information on the plant source in tabular form (Table S4), references, isolation process, structural elucidation, NMR, mass, and GC‐MS data, please refer to the Supporting Information.

### 2.1 Coronavirus 229E Assay

The protective effects of the samples against human coronavirus (HCoV) 229E were determined similarly to the previously described method [16, 17]. Details are in Supporting Information.

### 2.2 Pseudotyped Lentivirus Assay

Lentivirus experiments were approved by the Institutional Biosafety Committee of Chang Gung University and performed according to a previous report [18]. Cepharanthine served as a positive control. Details are in Supporting Information.

### 2.3 WST‐1 Viability Assay

The potential cytotoxicity of tested samples was evaluated by WST‐1 reduction assay in hACE‐2‐overexpressed HEK293T [18]. Details are in Supporting Information.

### 2.4 Molecular Docking

To evaluate the binding energy between **16** and SARS‐CoV2 S‐protein in the binding site with ACE2 receptor, molecular docking calculation was performed using Autodock 4.2 adopting a Lamarckian genetic algorithm [19]. For details, please refer to Supporting Information.

### 2.5 ChemGPS‐NP Analysis

The ChemGPS‐NP principal component analysis of alkaloids and reference anticoronavirus drugs was calculated using the online tool ChemGPS‐NPWeb (http://chemgps.bmc.uu.se) based on SMILES input from ChemBioDraw (version 17.0) or PubChem (https://pubchem.ncbi.nlm.nih.gov) [20]. Details in Supporting Information.

### 2.6 Drug‐Likeness and ADMET Predictions

The SMILES representation of each isolated compound was entered into prediction tools, and drug‐likeness was assessed based on the Lipinski Rule of Five [21, 22]. SwissADME [23] and pkCSM [24] were utilized to evaluate the pharmacokinetic characteristics and ADMET (absorption, distribution, metabolism, excretion, and toxicity) properties of the compounds.

### 2.7 Statistical Analysis

Results are expressed as the value of the mean of two independent measurements (coronavirus HCoV‐229E assay, calculated as means ± SD) after a single dose screening of 62 alkaloids (coronavirus HCoV‐229E assay, single measurement), and as means ± SEM of three independent measurements (pseudovirus neutralization assay). Comparisons were carried out using Student's *t* test (SigmaPlot, Jandel Scientific, San Rafael, CA, USA). Statistical significance was acceptable at a level of *p* < 0.05.

## 3 Results

This study screened IAs of various structural types, previously isolated within various phytochemical studies [25, 26, 27, 28], for in vitro anticoronavirus 229E activity. Moreover, active alkaloids were further explored by pseudovirus assays, molecular docking, and ChemGPS‐NP analysis to compare their chemical properties.

### 3.1 Protective Effects Against Human Coronavirus 229E Infection

Initially, the protective effects of alkaloids on the cells infected with HCoV‐229E in Huh7 cells were investigated (Table 1, Table S1, Figures S1 and S2), with results indicating that the most active compounds in the HCoV‐229E assay were alkaloids of haemanthamine type, heamanthidine (**1**) (EC_50_ 4.11 μM) isolated from *Zephyranthes robusta* (Amaryllidaceae), and a group of BBI alkaloids isolated from *Berberis vulgaris* (Berberidaceae), including aromoline **16** (EC_50_ 4.33 μM), berbamine **17** (EC_50_ 5.11 μM), bersavine **18** (EC_50_ 6.45 μM), and obamegine **19** (EC_50_ 8.07 μM).

**TABLE 1:** In vitro anticoronavirus 229E data of the most active alkaloids from a panel of 62 tested alkaloids.

Compound	Coronavirus
	HCoV‐229E, EC_50_ (μM)^a^
Haemanthidine (**1**)	4.11 ± 0.41
Aromoline (**16**)	4.33 ± 0.90
Berbamine (**17**)	5.11 ± 0.79
Bersavine (**18**)	6.45 ± 0.93
Obamegine (**19**)	8.07 ± 0.90

^a^ Concentration necessary for 50% of preventing cytopathic effect of HCoV‐229E infection in Huh7 cells, calculated as means ± SD (*n* = 2).

Homolycorine (**10**), muraricine (**20**), and berkristine (**21**) demonstrated modest protective effects against HCoV‐229E infection at 10 μM, with 40%, 50%, and 65% protection, respectively. Haemanthamine **3** and norchelidonine **48** showed 50% cytotoxicity but effectively protected all surviving cells from infection, indicating a narrow therapeutic window. Several alkaloids at 10 μM, including 6‐ethoxydihydrosanguinarine **51** and 6‐ethoxydihydrochelerythrine **52**, showed high cytotoxicity (> 80%) to the host cells, possibly due to the presence of the ethoxy functional group. Other alkaloids, such as thalictricavine **27**, scoulerine **33**, and chelidonine **50**, showed moderate toxicity (35%–40%). In contrast, anumber of compounds, including the active alkaloids **1** and **17**, demonstrated low cytotoxicity (~20%), indicating a more favorable safety profile at the tested concentration.

The literature indicates that several plant extracts and alkaloids possess anticoronavirus activity [29]. Thalimonine, a pavinane alkaloid from *Thalictrum simplex* L., exerted anti‐influenza activities by inhibiting viral reproduction, including the expression of glycoprotein haemagglutinin, neuraminidase, and nucleoprotein [30, 31]. However, in our study, the tested pavinane alkaloids (**23**, **24**, **34**, **35**, **36**) were inactive against HCoV‐229E infection. The most active IAs identified in this work were isolated from *B. vulgaris* L., a species well‐known for producing berberine, which has previously demonstrated antiviral activity against viruses such as hepatitis C virus, HPV, HIV, HSV, human cytomegalovirus (HCMV), Zika virus, enterovirus, and the influenza virus [32, 33, 34]. Interestingly, according to our results, 8‐oxoberberine showed no activity against the coronavirus 229E strain.

Thus, the promising protective effects of **1**, **16**, **17**, **18**, and **19** against HCoV‐229E virus infection were the motivation for further evaluation of their impact on the SARS‐CoV‐2 S‐protein/ACE2‐binding pseudovirus neutralization assay in ACE2‐expressing cells (ACE2‐293T).

### 3.2 SARS‐CoV‐2 Spike/ACE2 Pseudovirus Neutralization Assay

**FIGURE 1:**
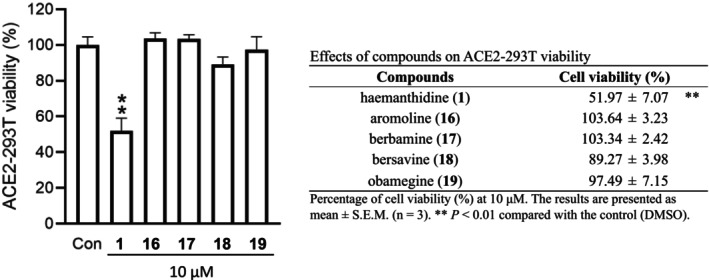
Viability assay of bis‐benzylisoquinoline alkaloids **1**, **16**–**19** in ACE2‐293T cells.

**FIGURE 2:**
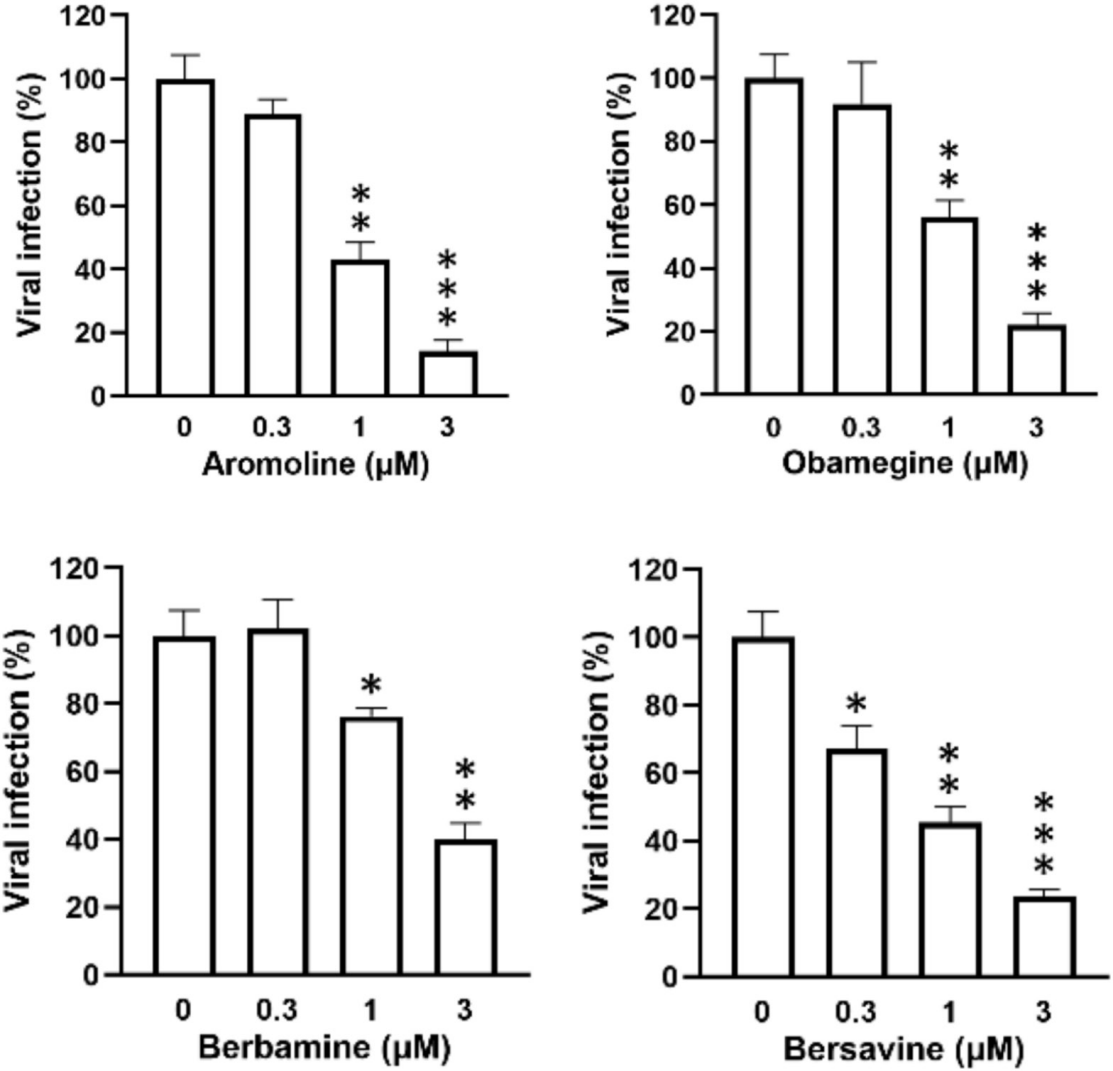
BBI alkaloids aromoline (**16**), berbamine (**17**), bersavine (**18**), and obamegine (**19**) inhibited the binding of SARS‐CoV‐2 S‐protein/ACE2 in a pseudovirus assay against Omicron strain infection (*n* = 3).

The S‐protein and ACE2 receptor are both essential in the early stages of coronavirus infection [35]. To assess this interaction, a binding assay was performed using stable hACE2‐overexpressed HEK293T cells and SARS‐CoV‐2 S‐protein expressing VSV‐G pseudotyped lentiviruses, with luciferase activity serving as the quantitative readout. In the following experiment, we selected three coronavirus subtypes: the SARS‐CoV‐2 variant carrying the S‐protein amino acid D614G mutations; the Delta variant representing the early strain; and the current variant, Omicron. It is well known that these mutations increase the infectivity of the COVID‐19 virus [6]. Firstly, the cytotoxicity towards hACE2‐293 T cells was evaluated, and the results revealed that haemanthamine type alkaloid **1** was toxic to the host cells (Figure 1), while in contrast, BBI‐type Compounds **16**, **17**, **18**, and **19** were nontoxic. The SARS‐CoV‐2 S‐protein and ACE2 binding assay results on different virus variants revealed a potent activity of **16**, **17**, **18**, and **19** (Figure 2, Table 2), while **1** was inactive at nontoxic concentration, indicating the different mechanisms of antiviral activity among the Amaryllidaceae and BBI alkaloids. In the pseudovirus neutralization assay, the most active was **16,** with IC_50_ values of 0.67 μM, 0.47 μM, and 0.86 μM against D614G, Delta, and Omicron variants, respectively. The coefficient of variation (approx. 10%) indicated higher but acceptable variability and significance. Compound **16** was significantly more effective than the positive control, E‐64 (IC_50_ 22.69–23.12 μM); interestingly, other BBI alkaloids **17**, **18**, and **19** were also active with IC_50_ values of 1.19–2.86 μM.

**TABLE 2:** Effects of compounds in the pseudovirus neutralization assay of D614G, Delta, and Omicron variants (SARS‐CoV‐2 spike protein pseudotyped lentivirus type).

Compounds	Pseudovirus assay, IC_50_ (μM)^a^		
	D614G	Delta	Omicron
Haemanthidine (**1**)	> 10	> 10	NT
Aromoline (**16**)	0.67 ± 0.09	0.47 ± 0.08	0.86 ± 0.12
Berbamine (**17**)	2.23 ± 0.49	2.56 ± 0.16	2.29 ± 0.29
Bersavine (**18**)	1.24 ± 0.17	2.86 ± 0.13	1.71 ± 0.34
Obamegine (**19**)	1.40 ± 0.35	2.61 ± 0.42	1.19 ± 0.16
E‐64	23.12 ± 0.63	23.06 ± 1.30	22.69 ± 1.28
Cepharantine	0.48 ± 0.09	1.64 ± 0.18	1.31 ± 0.07

^a^ Concentration necessary for 50% inhibition (IC_50_). Results are presented as mean ± SEM (*n* = 3). NT, not tested. E‐64 and cepharantine served as a positive control.

**FIGURE 3:**
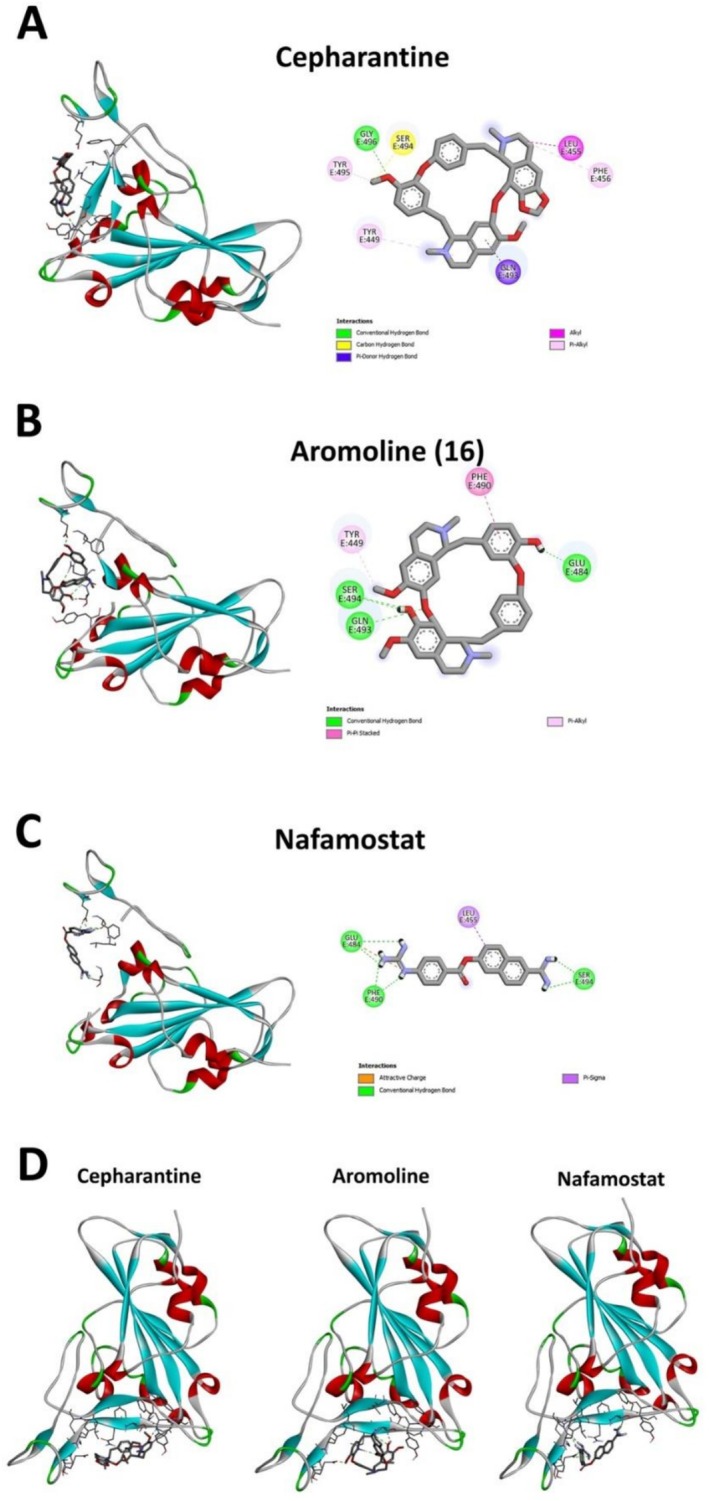
Molecular docking binding model of SARS‐CoV‐2 S‐protein with (A) cepharantine, (B) aromoline (**16**), and (C) inhibitor nafamostat. Left panel: The ligand (sticks) is positioned according to the best binding interaction with SARS‐CoV‐2 S‐protein. Green dash lines represent the hydrogen bonds between ligands and corresponding amino acids of the SARS‐CoV‐2 S‐protein binding site. Right panel: The amino acids' three‐letter abbreviations and numbers indicate the main residues contributing to the binding. Residues and dash lines in green, show classical hydrogen bonding; yellow, carbon–hydrogen bonding; blue, pi donor–hydrogen bonding; and color shades of red, hydrophobic bonding. The blue cloud represents solvent accessibility. Figure 3D illustrates the native position of SARS‐CoV‐2 S‐protein in the ACE2 binding pocket (bottom part).

Previous research suggested that reducing the activity of TMPRSS2 using plant secondary metabolites could help manage COVID‐19 [35]. TMPRSS2 is crucial for the virus entry stage by priming the S‐protein of SARS‐CoV‐2, which facilitates the fusion of viral and host cell membranes [4]. In an in silico molecular docking study of 4704 ligands with four target SARS‐CoV‐2 proteins, **16** interacted well with TMPRSS2 and, to a certain extent, with the SARS‐CoV‐2 S‐protein [36]. This data bodes well with the docking result for **16** and cepharantine (Figure 3) in the SARS‐CoV‐2 S‐protein/ACE2 binding pocket. Further, according to our results, E‐64, a TMPRSS2 inhibitor, showed weaker effects against the three SARS‐CoV‐2 strains (IC_50_ of 22.7–23.1 μM, Table 2). Similar to our results, a previous study found that BBI alkaloids, including cepharantine, blocked pseudovirus entry but did not specifically interact with ACE2. Instead, BBIs were proposed as pan‐coronavirus entry inhibitors that might abolish Spike–ACE2‐mediated membrane fusion by targeting the host calcium channel and suppressing virus entry [15].

### 3.3 Molecular Docking With SARS‐CoV‐2 S‐Protein

As indicated in the pseudovirus neutralization assay, the most active compound, **16**, efficiently inhibited the D614G, Delta, and Omicron variants compared to other isolated compounds and even positive controls (cepharantine and E‐64). Molecular docking was employed to further explore the binding efficiency of **16** to SARS‐CoV‐2 S‐protein as the SARS‐CoV‐2 receptor‐binding domain (RBD) of S‐protein and ACE2 has been identified and described previously [37], and the generated docking positioning indicated the interaction of ligands with the amino acid residues of the active site of S‐protein RBD (PDB ID: 6M0J) at the area of α‐helix between the Tyr449 and Tyr505 [38].

According to the docking results (Figure 3, Table 3), main hydrogen bonding interactions were formed with residues Glu484 and Gly496 for cepharantine (binding energy −5.56 kcal, inhibition constant Ki 84.5 μM), **Gln493** and Ser494 for aromoline (**16**) (−5.34 kcal, Ki 121.8 μM), and **Tyr453**, Phe490, Ser494 for nafamostat (−6.07 kcal, Ki 35.5 μM). Nafamostat is a human transmembrane serine protease (TMPRSS2) inhibitor that has been reported to inhibit the S‐protein binding and thus was used as a positive control [39]. Moreover, apart from the classical hydrogen bonds mentioned above, there were several other types of interactions observed for both cepharantine (Ser494 carbon–hydrogen, Gln 493 pi‐hydrogen, **Leu455** alkyl hydrophobic, Phe456, **Tyr449**, Tyr495 pi‐alkyl hydrophobic) and **16** (Glu484 hydrogen, Phe490 pi hydrophobic, Tyr449 pi‐alkyl hydrophobic interactions) (Table 3). Many of these interactions followed the reference binding interaction, such as Tyr449, Tyr453, Leu455, Gln493, Ser494, Glu484, Phe490, and Leu492 [38]; furthermore, the binding of both cepharantine or **16** directly to ACE2 was weak as indicated by relatively high binding energies (−4.91, H‐bond Lys353, Ki 253.0 μM; and −3.91, Lys353, Ki 1.35 mM respectively; data not shown). This indicated a better binding of cepharantine and **16** to the S‐protein of the virus rather than the unspecific binding to ACE2 on the human body's tissues.

**TABLE 3:** Binding energy and hydrogen bonds formed between SARS‐CoV‐2 S‐protein and ligand (cepharantine, aromoline, and S‐protein inhibitor nafamostat).

Target protein	Ligand	Binding energy (kcal)	Inhibition constant Ki (μM)	Hydrogen bonds	Number of hydrogen bonds
S‐protein	Cepharantine	−5.56	84.5	Glu484, Gly496	2
S‐protein	Aromoline (**16**)	−5.34	121.8	Gln493, Ser494	2
S‐protein	Nafamostat	−6.07	35.5	Tyr453, Phe490, Ser494	3

*Note:* S‐protein, SARS‐CoV‐2 spike protein, Protein Data Bank code 6M0J (http://www.rcsb.org), the S‐protein binding site to ACE2 was established (X:39.839390; Y:31.431017; Z:6.225943), box size (X:Y:Z, 40:40:40).

### 3.4 ChemGPS‐NP Chemical Space Analysis

**FIGURE 4:**
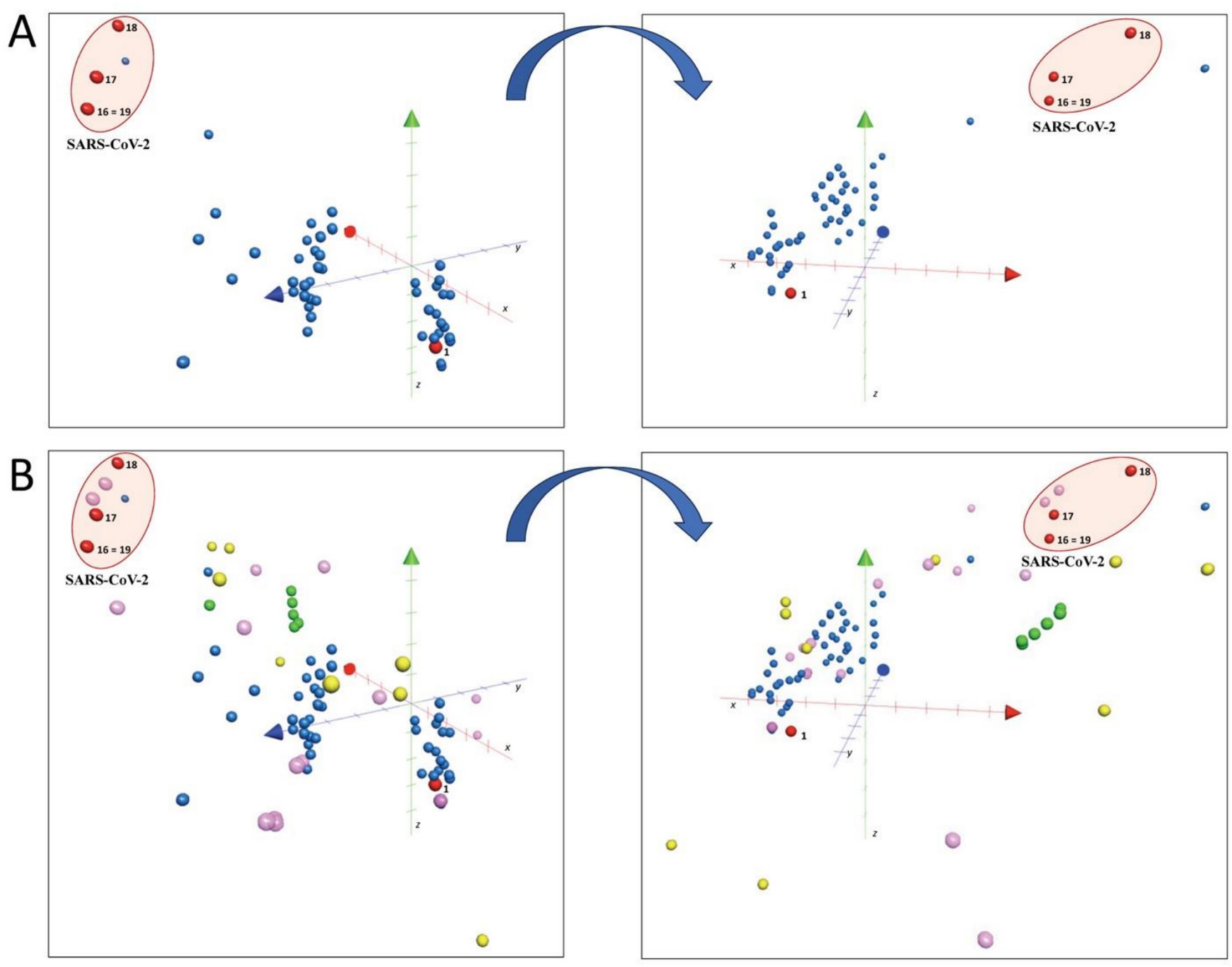
ChemGPS analysis of BBI (red active) with other tested (blue) alkaloids and herbal (pink), synthetic (green), and clinical (yellow) antiviral drugs. (A) Anticoronavirus 229E alkaloids (red dots; **1**, **16**, **17**, **18**, **19**) and inactive alkaloids (blue dots) were plotted alone or (B) together with anticoronavirus drugs (yellow chemical antivirotics, green indole derivatives, and pink natural alkaloid antivirotics). The compounds active in the SARS‐CoV‐2 Omicron assay are highlighted. The 3D plot is displayed as three principal component axes representing different physico‐chemical properties, that is, PC1 (red axis, *x*, size), PC2 (blue axis, *y*, aromaticity), and PC3 (green axis, *z*, lipophilicity) from two different angles.

Recently, computational analysis of physico‐chemical properties has often been used to correlate SARs. In silico modeling was performed, and alkaloids were plotted with synthetic and natural antiviral drugs using the chemical global positioning system for natural products (ChemGPS‐NP, Figure 4). It is a tool based on principal component analysis (PCA) that refers to a comprehensive and biologically relevant chemical space [40]. The physico‐chemical properties of compounds, composed initially of eight dimensions, were represented by the following properties: size, shape, and polarizability (PC1); aromaticity and conjugation‐related properties (PC2); and lipophilicity, polarity, and hydrogen bond capacity (PC3). The scores of these principal components (PC1, PC2, and PC3) were obtained based on SMILES of all compounds [40]. This method has been used to analyze the biological function of a series of natural product derivatives [41], including antiviral herbal isoflavonoids [42], anticancer dietary polyphenols [43], or marine anticancer secondary metabolites [44].

According to the results, the isolated alkaloids (blue dots) formed a pan‐blue cluster of two slightly separated sub‐clusters, the first representing Amaryllidaceae and the second other alkaloids (Figure 4A). The haemanthamine‐type alkaloid Compound **1,** active against HCoV‐229E coronavirus but toxic to the host cells and inactive against the Delta variant, was displayed at the edge of the pan‐blue cluster (**1**, red dot, lowest PC2 values, green axis). Interestingly, the active anti‐HCoV‐229E, anti‐Delta, and anti‐Omicron coronavirus BBI alkaloids (**16**, **17**, **18**, and **19**) formed a separate, well‐distinguished cluster (highlighted in red), located in the far corner of the chemical space with high values of PC1 and PC3, likely due to their dimeric nature. An in‐house database of reference drugs (Tables S2 and S3) that belong to the class of potential anticoronavirus medicines in different stages of development was also plotted, that is, clinical chemical antivirotics (yellow dots), indole derivatives (green dots), and natural alkaloid antivirotics (pink dots) (Figure 4B), as well as other natural product derivatives (Figure S3).

The active group of BBI alkaloids (**16**, **17**, **18**, and **19**, red dots) shared the aromaticity (PC2, green axis) and physico‐chemical properties with previously reported anticoronavirus alkaloids (pink dots; the closest were cepharanthine, 10‐hydroxyusambarensine, fangchinoline, and tetrandrine) [45]. Interestingly, the BBI anticoronavirus cluster (**16**–**19**) also shared PC1 dimensions (representing size and shape) with the α‐ketoamide indole derivatives (green dots) and some of the synthetic derivatives used in the clinic (yellow dots). Therefore, the results demonstrated common physico‐chemical properties of active BBI alkaloids (**16**, **17**, **18**, and **19**) shared with some of the known natural and clinical antivirotics with anticoronavirus potential that deserve further investigation.

Comparing spatial relationships between BBI alkaloids with antivirals, lopinavir showed the closest alignment based on values from PC1 and PC3, which indicate similarity in structural and physico‐chemical properties in chemical space. Meanwhile, ritonavir, which is typically co‐administered with lopinavir to improve pharmacokinetics, exhibited similarity only in PC3. This is opposite to remdesivir, which showed close value only in PC1, reflecting lower lipophilicity despite being one of the most widely used antivirals for COVID‐19.

In addition to their structural resemblance to known antivirals, BBIs offer complementary mechanisms, including multi‐target action, due to their capacity to inhibit both viral entry and host pathways. Combining BBI with clinical antivirals may enhance efficacy. For instance, BBIs (Compounds **16**–**19**, aromoline‐like) hold a scaffold that targets host receptors (e.g., ACE2) and viral proteases. Amarylidaceae alkaloids (e.g., haemanthidine‐like) consist of phenanthridone cores and C‐ring modifications that enhance RNA polymerase inhibition.

While clinically approved antivirals like Paxlovid (a combination of nirmatrelvir and ritonavir) and remdesivir can reduce hospitalization by up to 87%, their clinical use has certain limitations, including drug interactions (Paxlovid) and intravenous administration requirements (remdesivir) [46].

### 3.5 Drug‐Likeliness and Pharmacokinetics

The drug‐like properties of Compounds **1** and **16**–**19**, along with cepharanthine as a reference drug, were evaluated using Lipinski's Rule of Five for oral bioavailability and computational ADMET analysis for pharmacokinetic suitability [22, 47]. These properties are critical in determining the pharmacokinetic profile and oral bioavailability potential of candidate drug molecules.

Our ChemGPS analysis demonstrates that remdesivir and lycorine show similarities in chemical properties to active Compound **1** in chemical space. This finding is particularly significant given that *lycorine* from *Lycoris radiata* has previously been identified as potent against SARS‐CoV using a virus‐induced cytopathic effect assay [48].

Haemanthidine (**1**) met all of Lipinski's criteria, including molecular weight ≤ 500 Da, logP ≤ 5, hydrogen bond donors ≤ 5, hydrogen bond acceptors ≤ 10, and molar refractivity between 40 and 130, indicating good oral bioavailability (Table 4). Among the BBI alkaloids, Compounds **16**–**19**, including cepharanthine, satisfied two of Lipinski's criteria (hydrogen bond donors and acceptors), with aromoline (**16**) being the most promising candidate, coming closest to the recommended values, unlike the larger molecule, bersavine (**18**). It is important to note that Lipinski's guidelines are general recommendations rather than strict thresholds.

**TABLE 4:** Lipinski results of active compounds.

Compound	Molecular mass	Hydrogen bond donor	Hydrogen bond acceptor	Lipophilicity (LogP)	Molar refractivity	Complying with rule
Acceptable values	≤ 500 Da	≤ 5	≤ 10	≤ 5	Between 40 and 130	
**1**	**317.34**	**2**	**6**	**0.4449**	**79.324577**	**5**
**16**	594.71	**2**	**8**	6.556404	167.907806	2
**17**	608.74	**1**	**8**	6.859403	172.795044	2
**18**	693.89	**1**	**9**	7.7012	203.672	2
**19**	594.71	**2**	**8**	6.556404	167.907806	2
Cepharanthine	606.72	**0**	**8**	6.873902	170.701233	2

*Note:* The bolded compound **1** complies best with the Lipinski rules.

In the ADMET evaluation, aromoline (**16**) exhibited the most favorable pharmacokinetic profile. Aside from potential interactions with P‐glycoprotein and the CYP3A4 enzyme (Table 5), **16** showed good solubility, intestinal absorption, and excretion properties, along with a low toxicity profile, including no AMES mutagenicity and no hepatotoxicity. Notably, **16** outperformed the reference drug cepharanthine in several aspects, such as volume of distribution (VDss), renal OCT2 interaction (indicating lower nephrotoxicity risk), AMES mutagenicity, and chronic oral toxicity in rats.

**TABLE 5:** ADMET parameters of active compounds.

Property	Parameter (human)	Acceptable values	(±)‐Haemanthidine (1)	(+)‐Aromoline (16)	(+)‐Berbamine (17)	(+)‐Bersavine (18)	(+)‐Obamegine (19)	Cepharanthine
Absorption	Water solubility (log mol/L)	> −4	−1.611	**−2.901**	−2.901	−2.896	−3.108	−3.101
	Caco2 permeability (log Papp in 10–6 cm/s)	> 0.9	0.271	**1.046**	1.17	1.147	0.866	1.166
	Intestinal absorption (% absorbed)	> 80%	74.904	**92.024**	91.366	86.606	92.474	98.473
	Skin permeability (log Kp)	< −2.5	−2.848	**−2.735**	−2.735	−2.735	−2.735	−2.735
Distribution	P‐glycoprotein substrate (yes/no)	No	Yes	Yes	Yes	Yes	Yes	Yes
	P‐glycoprotein I inhibitor (yes/no)	No	No	Yes	Yes	Yes	Yes	Yes
	P‐glycoprotein II inhibitor (yes/no)	No	No	Yes	Yes	Yes	Yes	Yes
	VDss (log L/kg)	0.04–2	0.468	**−0.001**	−0.249	0.135	−0.729	−0.783
	Fraction unbound (Fu)	0.1–1	0.622	**0.385**	0.419	0.444	0.353	0.475
	BBB permeability (log BB)	> 0.3 (good) < −1 (poor)	−0.564	**−0.53**	−0.66	−0.722	−0.874	0.084
	CNS permeability (log PS)	> − 2	−2.785	−2.631	−2.691	−2.719	−2.634	−2.539
Metabolism	CYP2D6 substrate (yes/no)	No	No	**No**	No	No	No	No
	CYP3A4 substrate (yes/no)	No	No	Yes	Yes	Yes	Yes	Yes
	CYP1A2 inhibitor (yes/no)	No	No	**No**	No	No	No	No
	CYP2C19 inhibitor (yes/no)	No	No	**No**	No	No	No	No
	CYP2C9 inhibitor (yes/no)	No	No	Yes	No	Yes	No	No
	CYP2D6 inhibitor (yes/no)	No	No	**No**	No	No	No	No
	CYP3A4 inhibitor (yes/no)	No	No	**No**	No	Yes	No	No
Excretion	Total clearance (log mL/min/kg)	~0.5–1.5	0.965	**0.74**	0.734	0.782	0.784	0.779
	Renal OCT2 substrate (yes/no)	No	No	**No**	Yes	Yes	No	Yes
Toxicity	AMES toxicity (yes/no)	No	No	**No**	No	No	Yes	Yes
	Max. tolerated dose (log mg/kg/day)	< 0.5 (safe)	−0.189	**0.198**	−0.066	0.008	−0.209	0.135
	hERG I inhibitor (yes/no)	No	No	**No**	No	No	No	No
	hERG II inhibitor (yes/no)	No	No	Yes	Yes	Yes	Yes	Yes
	Oral rat acute toxicity (LD50, mol/kg)	> 2	2.927	**2.467**	2.46	2.458	2.508	3.111
	Oral rat chronic toxicity (LOAEL, log mg/kg_bw/day)	1	1.768	**2.325**	2.4	1.967	1.685	0.972
	Hepatotoxicity (yes/no)	No	Yes	**No**	No	No	No	No
	Skin sensitization (yes/no)	No	No	**No**	No	No	No	No
	*T. pyriformis* toxicity (log μg/L)	> –0.5	0.285	**0.285**	0.285	0.285	0.285	0.285
	Minnow toxicity (log mM)	> –0.3	2.857	−3.387	−5.68	−3.37	−1.507	−1.676

*Note:* The bolded compound **16** complies best with the recommended pharmacokinetic properties.

BBI alkaloids violate multiple Lipinski criteria, primarily due to high molecular weight, elevated lipophilicity, and excessive molar refractivity. However, this does not necessarily preclude their development as orally active agents, and this finding requires more proper interpretation [49]. Studies have demonstrated that natural products frequently break Lipinski rules [50]. A comprehensive analysis of 148 biologically active products available as drugs revealed that many successful therapeutic natural products violate traditional drug likeness filters [51]. Lipinski noted that the rule of five only holds for compounds that are not substrates for active transporters [49].

Our ADMET results support the statement that all BBI alkaloids are identified as P‐glycoprotein substrates, indicating active uptake routes that can compensate for unfavorable passive permeability. Transport mechanisms can overcome passive diffusion limitations imposed by high molecular weight and lipophilicity. This transporter‐facilitated entry pathway provides a plausible explanation for how large, lipophilic compounds can retain drug‐like behavior despite violating classical filters. Approximately 50% of orally administered new entities actually violate Lipinski's criteria yet demonstrate clinical success [52]. This further supports that strict adherence to Lipinski's rules may overlook valuable bioactive scaffolds. The rule's implicit assumption of passive diffusion as the primary cellular entry mechanism ignores the crucial role of transporters.

### 3.6 SAR

**FIGURE 5:**
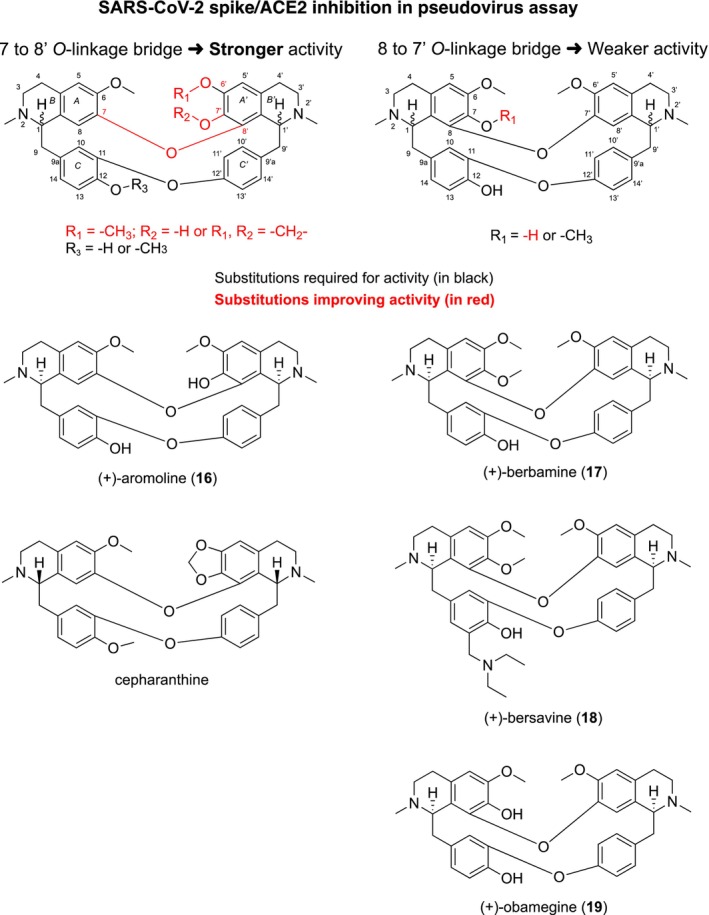
Structure–activity relationship of active BBI alkaloids (**16**–**19**, cepharanthine) in pseudovirus assay of D614G, Delta, and Omicron variants.

This in vitro study demonstrates that several bisbenzylisoquinoline (BBI) alkaloids—specifically aromoline, berbamine, bersavine, and obamegine—exhibit significant antiviral activity against both coronaviruses HCoV‐229E and SARS‐CoV‐2 (Table 1 and Figures 1 and 2). These findings align with the previous reports identifying other BBI alkaloids, such as cepharanthine, hernandezine, tetrandrine, and neferine as pan‐coronavirus entry inhibitors. These compounds protect various cell lines (ACE2‐293T, Calu‐3, and A549) from infection by different coronaviruses (SARS‐CoV, MERS‐CoV, SARS‐CoV‐2, including variants S‐D614, S‐G614, and N501Y) by blocking host calcium channels, thereby inhibiting Ca^2+^‐mediated fusion and virus entry [15]. The antiviral effects observed for the BBI and AAs in HCoV‐229E (Table 1) align with these earlier findings. In particular, the BBI Compounds **16** to **19** appear to inhibit the entry of SARS‐CoV‐2 pseudoviruses into hACE2‐overexpressing cells, potentially through interference with spike protein/ACE2 binding. For example, BBI cepharantine was previously reported to bind to the S‐protein and modulate ACE2 activation [53]. The SAR of different types of BBI alkaloids drawn from the current study is illustrated in Figure 5.

All bioactive BBIs share a characteristic phenolic *o*‐linkage connecting each two benzylisoquinoline units via head‐to‐head and tail‐to‐tail ether bonds. SAR analysis highlights the importance of both the position of phenolic *O*‐linkages and the substitution pattern of the methoxy or hydroxy groups. Specifically, Compound **16**, which features hydroxy groups at positions 12 and 7′ and an *O*‐linkage from 7 to 8′, demonstrated high antiviral activity. In contrast, Compounds **17**–**19** lacking the 7′ hydroxy group and instead bearing an *o*‐linkage bridge from 8 to 7′, showed slightly reduced potency. The presence of a diethylaminomethyl group at C‐15 in Compound **18** did not significantly influence activity, suggesting it is not a key pharmacophore.

Comparison with cepharanthine, a known antiviral BBI alkaloid, showed that Compounds **16**–**19** achieved comparable IC_50_ values (0.48–1.64 μM) against all three strains. Notably, cepharantine was more effective against D614G but less effective than **16** against Delta and Omicron. Although both cepharantine and **16** share the same 7 to 8′ phenolic *o*‐linkage bridge, cepharantine additionally features a 6′,7′‐dioxolane ring (Figure 5). This suggests that the presence of dioxolane moiety and specific stereochemistry at positions 1 and 1′ do not significantly impact spike‐ACE2 interaction in the pseudovirus assay.

## 4 Conclusion

In summary, BBI‐type alkaloids such as aromoline (**16**), obamegine (**19**), bersavine (**18**), and berbamine (**17**) demonstrated significant anticoronavirus activities. The findings from the coronavirus 229E and coronavirus pseudovirus assay highlight aromoline (**16**) as a promising candidate for future coronavirus disease management. Additionally, ChemGPS‐NP analysis suggests that BBI alkaloids possess suitable physico‐chemical properties for antiviral drug development targeting the entry stage of the virus cycle.

## Author Contributions

**Marcela Safratova:** data curation, formal analysis, writing – original draft, writing – review and editing, investigation, resources, conceptualization. **Yu–Li Chen:** methodology, formal analysis, validation, data curation, investigation, writing – original draft, writing – review and editing. **Anna Hostalkova:** writing – original draft, writing – review and editing, investigation. **Jakub Chlebek:** writing – review and editing, writing – original draft, investigation. **Chung–Fan Hsieh:** writing – original draft, writing – review and editing, investigation. **Bing–Hung Chen:** writing – review and editing, writing – original draft. **Lucie Cahlikova:** supervision, writing – review and editing, writing – original draft, resources. **Stefan Kosturko:** writing – original draft, writing – review and editing, investigation. **Anders Backlund:** investigation, validation, formal analysis, software, writing – review and editing, writing – original draft. **Jim–Tong Horng:** writing – original draft, writing – review and editing, methodology, validation, formal analysis, visualization, investigation. **Tsong–Long Hwang:** supervision, project administration, methodology, funding acquisition, writing – original draft, writing – review and editing, resources. **Michal Korinek:** project administration, data curation, validation, investigation, conceptualization, writing – original draft, writing – review and editing, formal analysis.

## Consent

The authors have nothing to report.

## Conflicts of Interest

The authors declare no conflicts of interest.

## Peer Review

The peer review history for this article is available at https://www.webofscience.com/api/gateway/wos/peer‐review/10.1111/irv.70166.

## Supporting information


**Scheme S1** Structures of isoquinoline alkaloids 1–62.
**Table S1:** In vitro anticoronavirus 229E screening data of the panel of alkaloids.
**Figure S1:** In vitro anticoronavirus 229E screening data of the panel of 62 alkaloids (compounds 1–62, n = 1).
**Figure S2:** In vitro anticoronavirus 229E data and EC50 calculation evaluation of active alkaloids 1, 16–19 screened from the panel of 62 alkaloids (n = 2).
**Figure S3:** ChemGPS‐NP analysis of 62 alkaloids correlated to anticoronavirus drugs, including natural products (alkaloids, terpenes, flavonoids, and other phenolics).
**Table S2:** ChemGPS‐NP data of the isoquinoline alkaloids 1–62 (available in Excel format).
**Table S3:** ChemGPS‐NP data of in‐house database for anticoronavirus drugs (available in Excel format).
**Table S4:** Compounds and their sources.

## Data Availability

The data supporting this study's findings are available in the manuscript and Supporting Information of this article.
