# Supplementary material for: Anticoronavirus Isoquinoline Alkaloids: Unraveling the Secrets of Their Structure–Activity Relationship
Source: Influenza Other Respir Viruses. 2025 Oct 5;19(10):e70166. doi: 10.1111/irv.70166 (PMC12497685; doi:10.1111/irv.70166)
Supplement: Supplementary file 1 — Scheme S1 Structures of isoquinoline alkaloids 1–62. Table S1: In vitro anticoronavirus 229E screening data of the panel of alkaloids. Figure S1: In vitro anticoronavirus 229E screening data of the panel of 62 alkaloids (compounds 1–62, n = 1). Figure S2: In vitro anticoronavirus 229E data and EC50 calculation evaluation of active alkaloids 1, 16–19 screened from the panel of 62 alkaloids (n = 2). Figure S3: ChemGPS‐NP analysis of 62 alkaloids correlated to anticoronavirus drugs, including natural products (alkaloids, terpenes, flavonoids, and other phenolics). Table S2: ChemGPS‐NP data of the isoquinoline alkaloids 1–62 (available in Excel format). Table S3: ChemGPS‐NP data of in‐house database for anticoronavirus drugs (available in Excel format). Table S4: Compounds and their sources. [file IRV-19-e70166-s001.pdf]

Supporting Information

Anti-coronavirus isoquinoline alkaloids: unraveling the secrets of their structure-activity relationship.

Short title: SAR of anti-coronavirus isoquinoline alkaloids

Marcela Safratova[a#], Yu-Li Chen[b,c#], Anna Hostalkova[a], Jakub Chlebek[a], Chung-Fan Hsieh[d,e], Bing-Hung Chen[f], Lucie Cahlikova[a], Stefan Kosturko[a], Anders Backlund[g], Jim-Tong Horng[h], Tsong-Long Hwang[b,c,i,j*], Michal Korinek[i,k,l,m*]

[a]*Department of Pharmacognosy and Pharmaceutical Botany, Faculty of Pharmacy, Charles University, 50005 Hradec Kralove, Czech Republic*

[b]*Center for Drug Research and Development, College of Human Ecology, Chang Gung University of Science and Technology, Taoyuan 33303, Taiwan*

[c]*Graduate Institute of Health Industry Technology, College of Human Ecology, Chang Gung University of Science and Technology, Taoyuan 33303, Taiwan*

[d]*Research Center for Emerging Viral Infections, College of Medicine, Chang Gung University, Taoyuan 33302, Taiwan*

[e]*Molecular Infectious Disease Research Center, Chang Gung Memorial Hospital, Chang Gung University College of Medicine, Taoyuan 333, Taiwan*

[f]*Department of Biotechnology, College of Life Science, Kaohsiung Medical University, Kaohsiung 80708, Taiwan*

[g]*Department of Pharmaceutical Biosciences, Faculty of Pharmacy, Uppsala University, Sweden*

[h]*Department of Biochemistry and Molecular Biology, College of Medicine, Chang Gung University, Kweishan, Taoyuan 33302, Taiwan*

[i] *Graduate Institute of Natural Products, College of Medicine, Chang Gung University, Taoyuan 333, Taiwan*

[j] *Department of Anesthesiology, Chang Gung Memorial Hospital, Taoyuan 33305, Taiwan*

[k] *Graduate Institute of Natural Products, College of Pharmacy, Kaohsiung Medical University, Kaohsiung 807, Taiwan*

[l] *Department of Medical Research, Kaohsiung Medical University Hospital, Kaohsiung Medical University, Kaohsiung, Taiwan*

[m] *Drug Development and Value Creation Research Center, Kaohsiung Medical University, Kaohsiung, Taiwan*

[#] Authors contributed equally to this work

Correspondence:

* **Tsong-Long Hwang**, Center for Drug Research and Development and Graduate Institute of Health Industry Technology, College of Human Ecology, Chang Gung University of Science and Technology, No. 261, Wenhua 1st Rd., Guishan Dist., Taoyuan, 33302, Taiwan. Tel & Fax: 886-3-2118506, ORCID: 0000-0002-5780-3977 (T.-L.H.). E-mail: htl@mail.cgust.edu.tw

* **Michal Korinek**, Graduate Institute of Natural Products, College of Pharmacy, Kaohsiung Medical University, 100 Shih-Chuan 1st Road, Kaohsiung 807, Taiwan. Tel: +886-7-3121101, ext. 2197. Fax: +886-7-3114773. ORCID: 0000-0002-8988-8610. E-mail: michalk@kmu.edu.tw

Table of Contents

Scheme S1. Structures of isoquinoline alkaloids 1-62 .... S4-S7

Table S1. In vitro anti-coronavirus 229E screening data of the panel of alkaloids S8

Figure S1. In vitro anti-coronavirus 229E screening data of the panel of 62 alkaloids (compounds 1-62, n = 1). S9

Figure S2. In vitro anti-coronavirus 229E data and EC_{50} calculation evaluation of active alkaloids 1, 16-19 screened from the panel of 62 alkaloids (n = 2) S10

Figure S3. ChemGPS-NP analysis of 62 alkaloids correlated to anti-coronavirus drugs, including natural products (alkaloids, terpenes, flavonoids, and other phenolics) S11

Table S2. ChemGPS-NP data of the isoquinoline alkaloids 1-62 (available in Excel format) S12

Table S3. ChemGPS-NP data of in-house database for anti-coronavirus drugs (available in Excel format) S13

Materials and Methods S14

Table S4. Compounds and their sources S14

2.1 Coronavirus 229E assay S15

2.2 Pseudotyped lentivirus assay S15

2.3 WST-1 viability assay S16

2.4 Molecular docking S16

2.5 ChemGPS-NP analysis S16

2.6 Statistical analysis S17

References S17

Amaryllidaceae alkaloids

Haemanthamin type

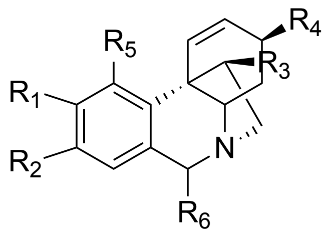

1: $R_1 + R_2 = OCH_2O$, $R_3 = OH$, $R_4 = OCH_3$, $R_5 = H$, $R_6 = OH$
2: $R_1 + R_2 = OCH_2O$, $R_3 = OH$, $R_4 = OH$, $R_5 = H$, $R_6 = H$
3: $R_1 + R_2 = OCH_2O$, $R_3 = OH$, $R_4 = OCH_3$, $R_5 = H$, $R_6 = H$
6: $R_1 = OCH_3$, $R_2 = H$, $R_3 = H$, $R_4 = O$, $R_5 = OH$, $R_6 = H$

Tazzetin type

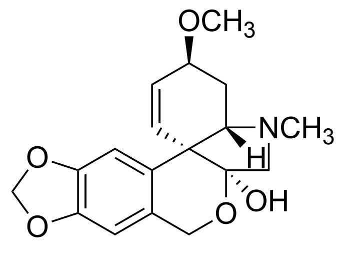

5

Homolycorine type

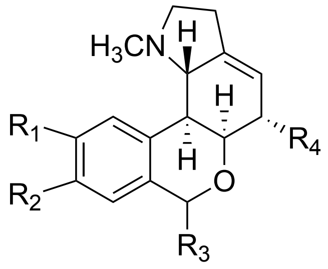

7: $R_1 = OCH_3$, $R_2 = OCH_3$, $R_3 = OH$, $R_4 = H$
8: $R_1 + R_2 = OCH_2O$, $R_3 =OH$**,** $R_4 = H$
10: $R_1 = OCH_3$, $R_2 = OCH_3$, $R_3 = O$, $R_4 = H$
11: $R_1 + R_2 = OCH_2O$, $R_3 =O$**,** $R_4 = H$
12: $R_1 + R_2 = OCH_2O$, $R_3 =O$**,** $R_4 = OH$

Lycorine type

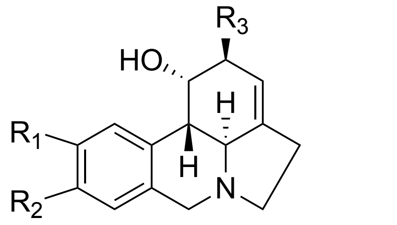

9: $R_1 + R_2 = OCH_2O$, $R_3 = OH$
4: $R_1 = OH$, $R_2 = OCH_3$, $R_3 = HOCH_3$
15: $R_1 + R_2 = OCH_2O$, $R_3 = H$

Crinine type

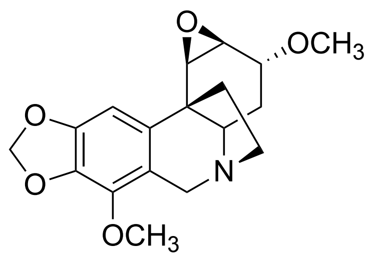

13

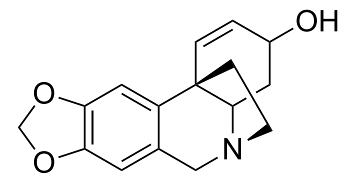

14

Scheme S1. Structures of isoquinoline alkaloids **1-62**.

Other isoquinoline alkaloids

Bis-benzylisoquinoline type

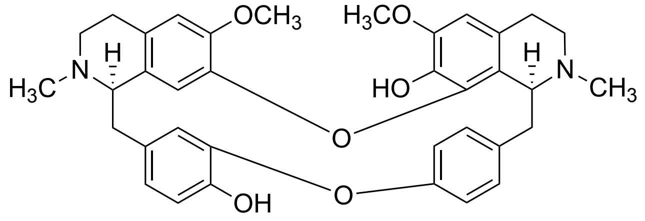

16

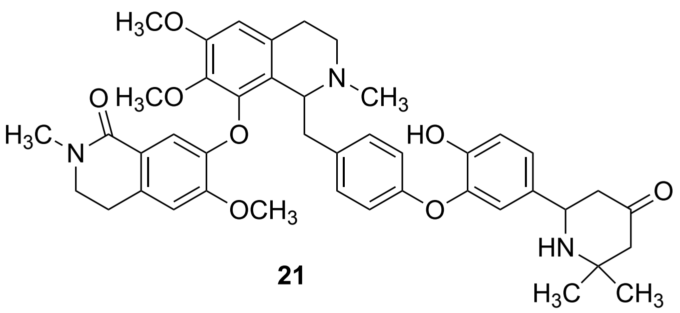

21

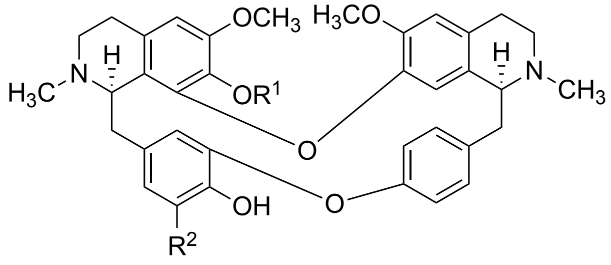

17: $R_1 = CH_3$, $R_2 = H$
18: $R_1 = CH_3$, $R_2 = CH_2N(CH_2CH_3)_2$
19: $R_1 = H$, $R_2 = H$

Benzylisoquinoline type

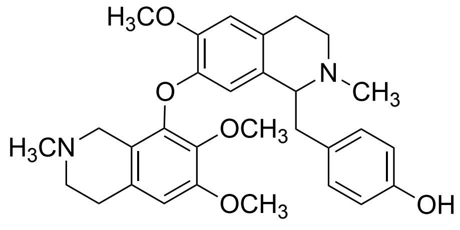

20

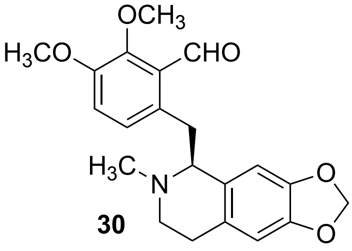

30

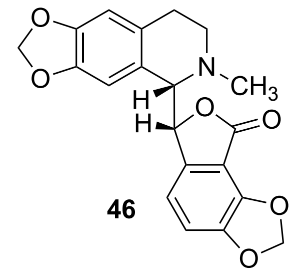

46

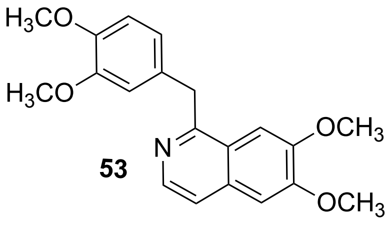

53

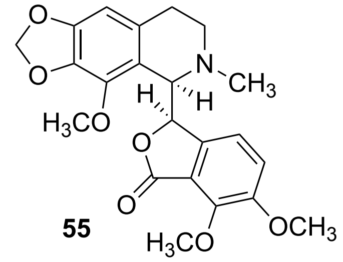

55

Scheme S1. (contd.) Structures of isoquinoline alkaloids **1-62**.

Protoberberine type

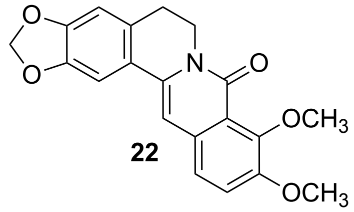

22

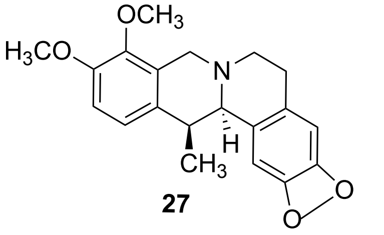

27

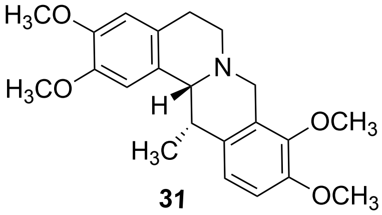

31

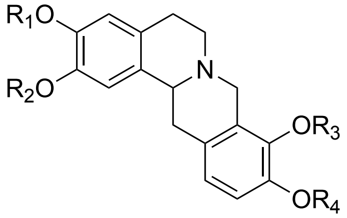

25: R_1 = H, R_2 = CH_3, R_3 = CH_3, R_4 = CH_3
26: R_1 + R_2 = CH_2, R_3 = CH_3, R_4 = CH_3
32: R_1 = CH_3, R_2 = H, R_3 = CH_3, R_4 = CH_3
33: R_1 = CH_3, R_2 = H, R_3 = H, R_4 = CH_3
43: R_1 = CH_3, R_2 = H, R_3 + R_4 = CH_2
49: R_1 + R_2 = CH_2, R_3 + R_4 = CH_2

Pavinane type

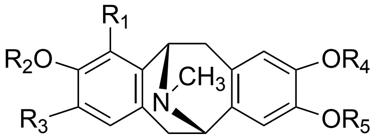

23: R_1 = H, R_2 = CH_3, R_3 = OCH_3, R_4 = CH_3, R_5 = CH_3
24: R_1 = OH, R_2 = CH_3, R_3 = H, R_4 = CH_3, R_5 = CH_3
34: R_1 = H, R_2 = H, R_3 = OCH_3, R_4+ R_5 = CH_2
35: R_1 = H, R_2 = CH_3, R_3 = OCH_3, R_4+ R_5 = CH_2
36: R_1 = OCH_3, R_2 = CH_3, R_3 = H,R_4+ R_5 = CH_2

Protopine type

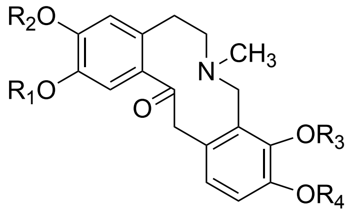

29; R_1 + R_2 = CH_2, R_3, R_4 = CH_3
45; R_1, R_2 = CH_3, R_3 + R_4 = CH_2

Indenobenzazepine type

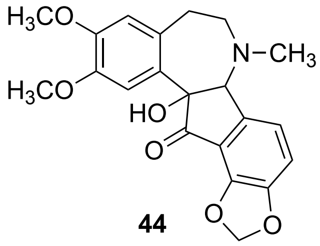

44

Scheme S1. (contd.) Structures of isoquinoline alkaloids **1-62**.

Aporphine type

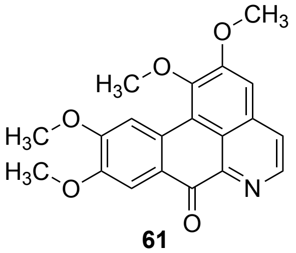

61

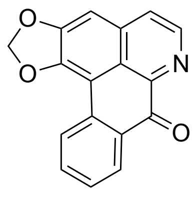

62

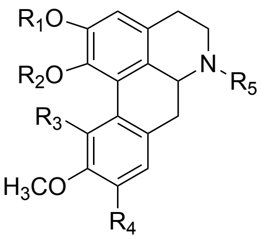

28: $R_1 + R_2 = CH_2$, $R_3 = OH$, $R_4 = H$, $R_5 = CH_3$
47: $R_1 = CH_3$, $R_2 = CH_3$, $R_3 = H$, $R_4 = OCH_3$, $R_5 = CH_3$
56 :$R_1 = CH_3$, $R_2 = CH_3$, $R_3 = H$, $R_4 = OH$, $R_5 = CH_3$
57 :$R_1 = CH_3$, $R_2 = CH_3$, $R_3 = OH$, $R_4 = H$, $R_5 = H$
58: $R_1 = CH_3$, $R_2 = CH_3$, $R_3 = H$, $R_4 = OH$, $R_5 = H$

Spirobenzylisoquinoline type

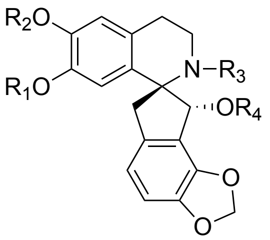

37; $R_1 = H$, R_2, $R_3 = CH_3$, $R_4 = COCH_3$
38; R_1, R_2, $R_3 = CH_3$, $R_4 = H$
39; R_1, R_2, $R_3 = CH_3$, $R_4 = COCH_3$
40; $R_1 = H$, R_2, $R_3 = CH_3$, $R_4 = H$

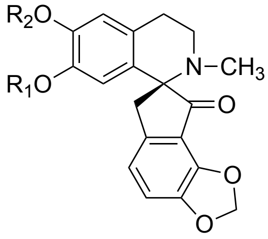

41; $R_1 + R_2 = CH_2$
42; $R_1 = H$, $R_2 = CH_3$

Benzophenanthridine type

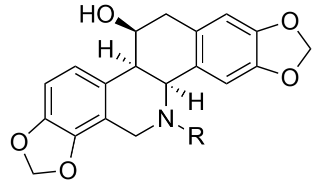

48; $R = H$
50; $R = CH_3$

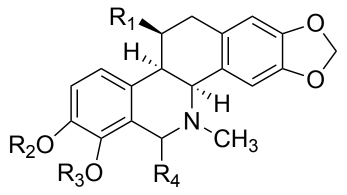

51; $R_1 = H$, $R_2 + R_3 = CH_2$, $R_4 = OCH_2CH_3$
52; $R_1 = OCH_2CH_3$, R_2, $R_3 = CH_3$, $R_4 = H$

Morphine type

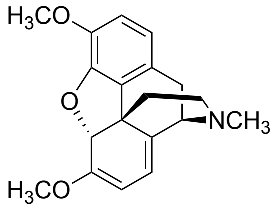

54

Homomorfinane type

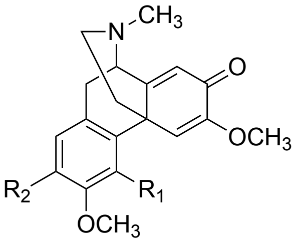

59; $R_1 = OH$, $R_2 = H$
60; $R_1 = H$, $R_2 = OH$

Scheme S1. (contd.) Structures of isoquinoline alkaloids **1-62**.

Table S1. In vitro anti-coronavirus 229E screening data of the panel of alkaloids.

Compound	Protective effects HCoV-229E % [a]	Cytotoxicity to the host Huh7 cells % [b]	Compound	Protective effects HCoV-229E % [a]	Cytotoxicity to the host Huh7 cells % [b]	Compound	Protective effects HCoV-229E % [a]	Cytotoxicity to the host Huh7 cells % [b]
haemanthidine (**1**)	**80%**	20%	8-oxoberberine (**22**)	–	–	sinactine (**43**)	–	–
hamayne (**2**)	–	–	argemonine (**23**)	–	–	*O*-methylfumarofine (**44**)	–	–
haemanthamine (**3**)	50%	50%	platycerine (**24**)	–	–	cryptopine (**45**)	–	–
9-*O*-demethylgalanthine (**4**)	–	–	corypalmine (**25**)	–	–	bikukuline (**46**)	–	–
tazzetine (**5**)	–	–	canadine (**26**)	–	–	glaucine (**47**)	–	–
seco-isopowellaminone (**6**)	–	20%	thalictricavine (**27**)	–	35%	norchelidonine (**48**)	50%	50%
lycorenine (**7**)	–	20%	bulbocapnine (**28**)	–	–	stylopine (**49**)	–	–
oduline (**8**)	–	20%	allocryptopine (**29**)	–	–	chelidonine (**50**)	–	35%
lycorine (**9**)	–	–	canadaline (**30**)	–	–	6-ethoxydihydrosanguinarine (**51**)	–	90%
homolycorine (**10**)	40%	–	corydaline (**31**)	–	–	6-ethoxydihydrochelerythrine (**52**)	–	80%
masonine (**11**)	–	–	tetrahydrocolumbamine (**32**)	–	–	papaverine (**53**)	–	20%
hippeastrine (**12**)	–	25%	scoulerine (**33**)	–	40%	thebaine (**54**)	–	–
undulatine (**13**)	–	–	caryachine (**34**)	–	–	narcotine (**55**)	–	20%
crinine (**14**)	–	–	*O*-methylcaryachine (**35**)	–	–	*N*-methyllaurotetanine (**56**)	–	–
caranine (**15**)	–	–	*O*-methylneocaryachine (**36**)	–	–	norisocorydine (**57**)	–	–
aromoline (**16**)	**85%**	–	fumarofycine hydrochloride (**37**)	–	–	laurotetanine (**58**)	–	–
berbamine (**17**)	**70%**	25%	fumaricine (**38**)	–	–	salutaridine (**59**)	–	–
bersavine (**18**)	**90%**	–	*O*-methylfumarofycine (**39**)	–	–	pallidine (**60**)	–	–
obamegine (**19**)	**80%**	–	fumaritine (**40**)[c]	–	–	oxoglaucine (**61**)	–	–
muraricine (**20**)	50%	–	fumariline (**41**)	–	–	liriodenine (**62**)	–	–
berkristine (**21**)	65%	–	parfumine (**42**)	–	–			–

[a]Percentage (%) of protective effects from HCoV-229E infection at 10 μM, compared with vehicle control (n = 1). — Not active (insignificant protective effects below 20%)
[b]Percentage (%) of cytotoxicity to host Huh7 cells at 10 μM compared with vehicle control (n = 1). — Non-toxic (insignificant cytotoxic effects, below 20%)
[c]The sample was tested at 1 µM due to low solubility in DMSO.

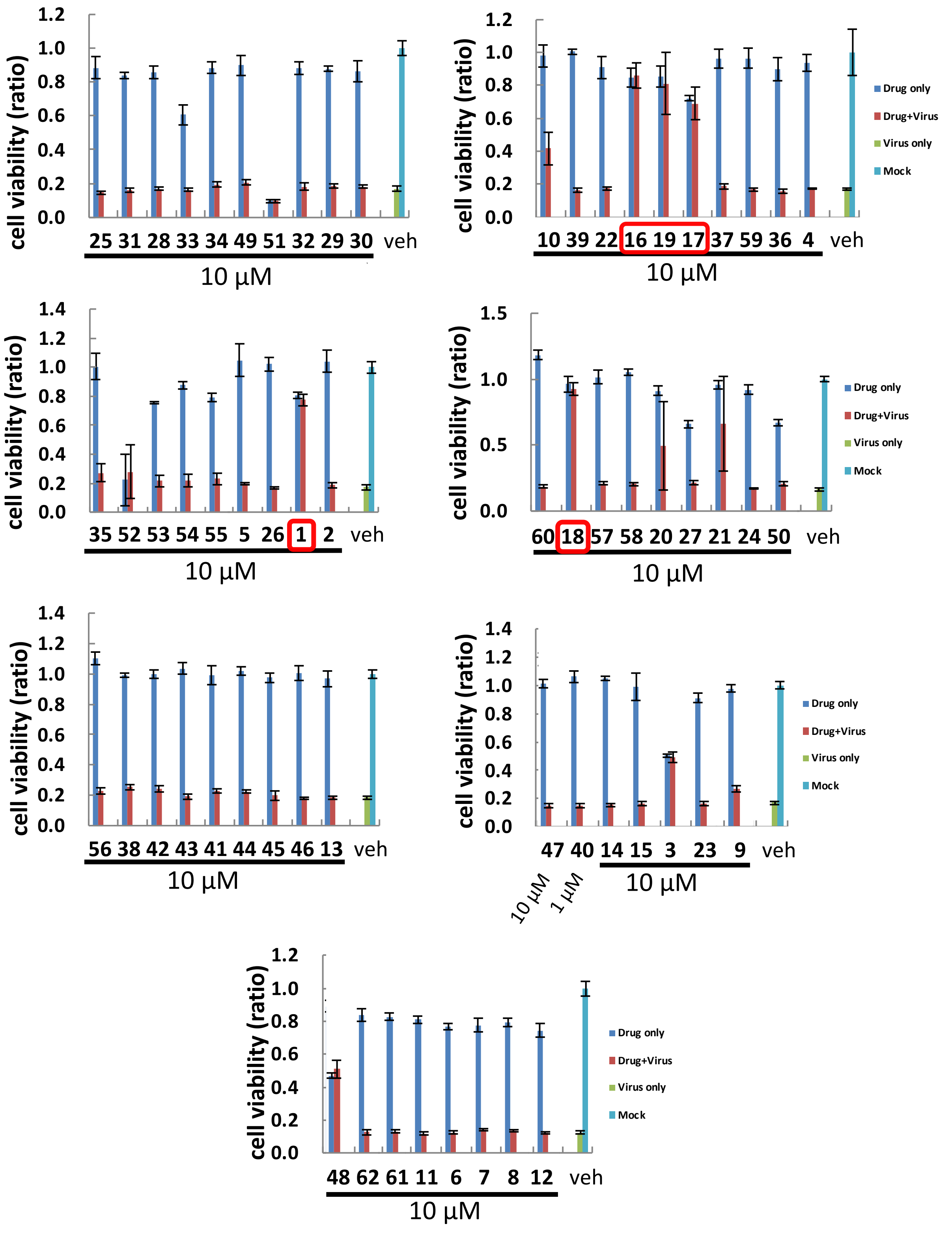

Figure S1. In vitro anti-coronavirus 229E screening data of the panel of 62 alkaloids (compounds **1**-**62**, n = 1). Protective effects against HCoV-229E infection. The Huh7 cells were infected with HCoV-229E and treated with either the samples (red) or vehicle control (green). A higher viability in the sample-treated group compared to the control indicates a protective effect against viral infection. In parallel, uninfected Huh7 cells were treated with the same samples (dark blue) or vehicle (light blue) to assess toxicity. A significant drop in cell viability in the sample-treated group relative to the control would indicate cytotoxicity. Huh7 cells, human liver carcinoma cell line. Vehicle, veh.

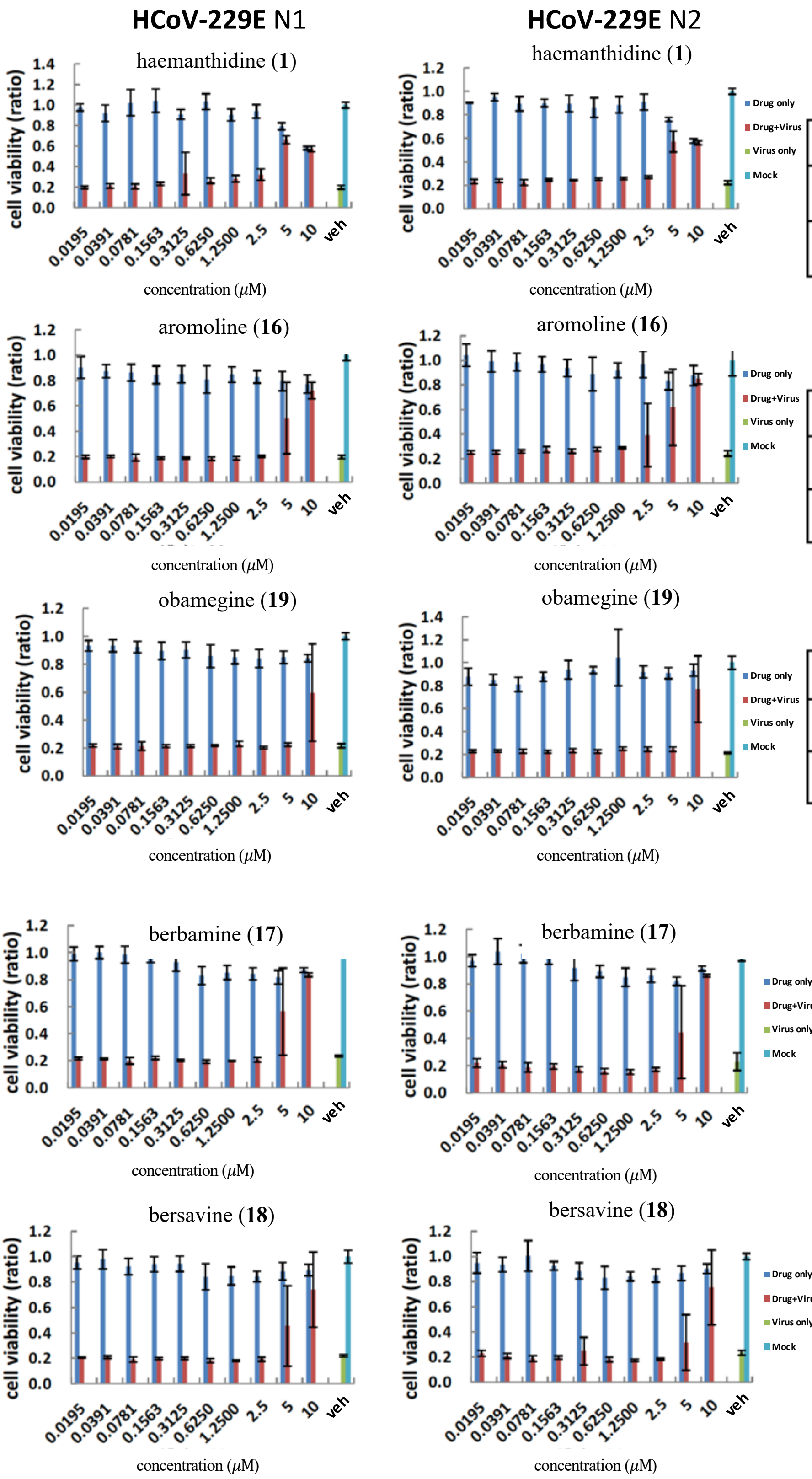

haemanthidine (**1**)

N	EC_{50} (μM)
1	3.820075188
2	4.405563033

aromoline (**16**)

N	EC_{50} (μM)
1	4.970053767
2	3.691031689

obamegine (**19**)

N	EC_{50} (μM)
1	8.701652847
2	7.430171221

berbamine (**17**)

N	EC_{50} (μM)
1	4.550120278
2	5.665708812

bersavine (**18**)

N	EC_{50} (μM)
1	5.792741165
2	7.104696983

Figure S2. In vitro anti-coronavirus 229E data and EC_{50} calculation of active alkaloids **1**, **16-19** screened from the panel of 62 alkaloids (n = 2). Vehicle, veh.

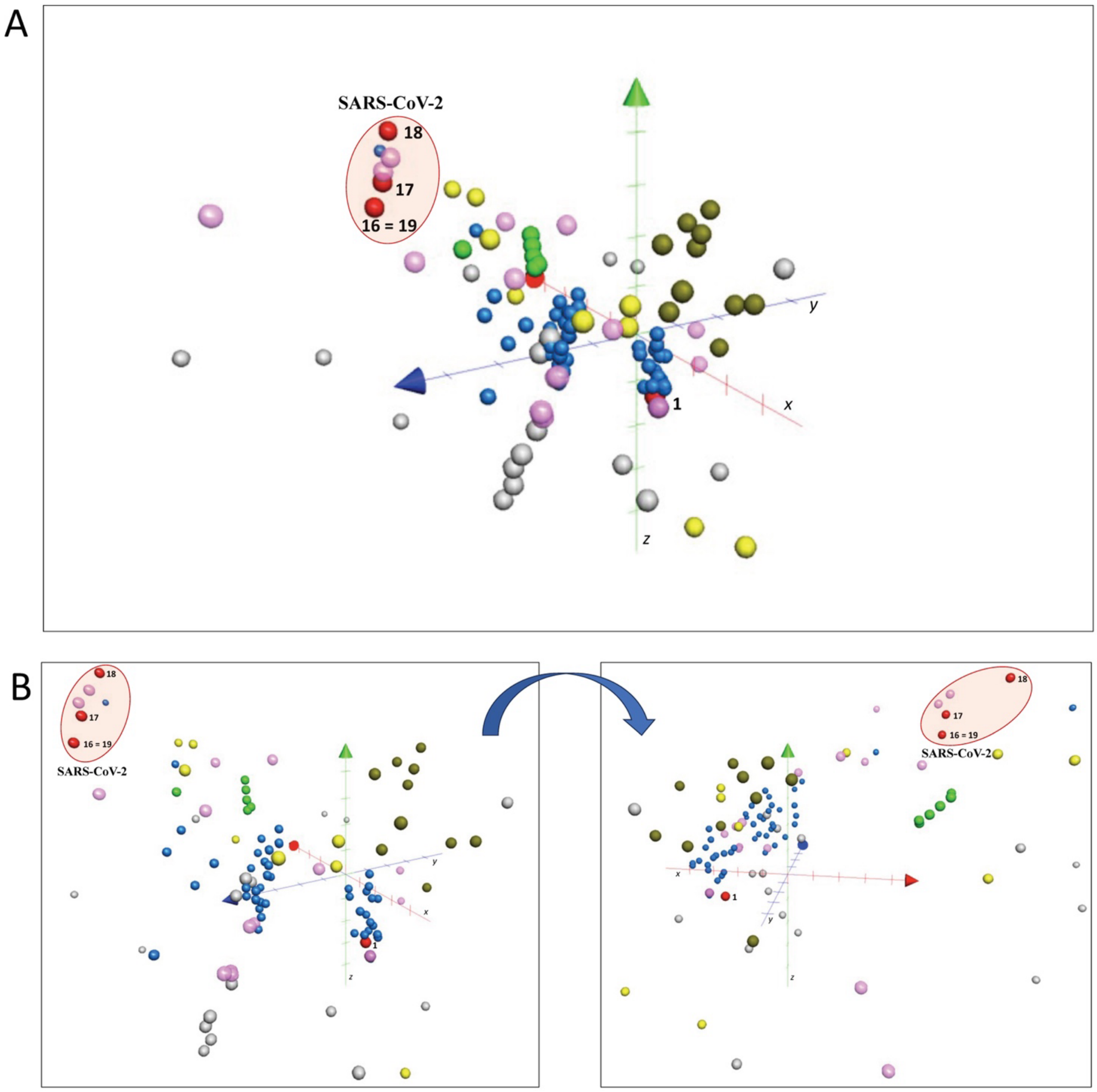

Figure S3. **ChemGPS-NP analysis of 62 alkaloids correlated to anti-coronavirus drugs, including natural products (alkaloids, terpenes, flavonoids, and other phenolics).** (**A**) Overview and (**B**) details with a view from the other side. Anti-coronavirus 229E alkaloids (red dots, ●; **1**, **16**, **17**, **18**, **19**), or inactive alkaloids (blue dots, ●) were plotted together with anti-coronavirus drugs including chemical antivirotics (yellow dots, ●), indole derivatives (green dots, ●) and natural alkaloids (pink dots, ●), natural terpenes (dark green dots, ●) and other natural compounds with anti-coronavirus activities (grey dots, ●). The 3D plot is displayed as three principal component axes representing different physico-chemical properties, i.e., PC1 (red axis ➔; *x*; size), PC2 (blue axis ➔; *y*; aromaticity), and PC3 (green axis ➔; *z*; lipophilicity).

Table S2. ChemGPS-NP data of the isoquinoline alkaloids **1-62** (available in Excel format)

Alkaloids isolated from various plant materials: inactive (●) or anti-SARS-CoV-2 (●)

Cpd.	*Plant source*		*Compound name*	*SMILES (ChemDraw 20.1)*	**ChemGPS-NP data** PC1 (➔)	PC2 (➔)	PC3 (➔)	PS4	PS5	PS6	PS7	PS8
**14**	*Nerine bowdenii*	●	(-)-crinine	OC1CC2N3CC4=CC5=C(OCO5)C=C4C2(CC3)C=C1	-2.224466	0.801391	-0.17817	-1.173	-0.003	1.2349	-0.188	0.357
**15**	*Nerine bowdenii*	●	(-)-caranine	OC1CC=C(CC2)C3N2CC4=CC5=C(OCO5)C=C4C31[H]	-2.223722	0.812213	-0.12802	-1.16	-0.015	1.2284	-0.208	0.3327
**6**	*Narcissus* cv. Dutch Master	●	seco-isopowellaminone	O=C1CC2N3CC4=CC=C(OC)C(O)=C4C2(CC3)C=C1	-2.139514	0.995271	-0.12201	-0.752	0.2719	0.817	0.0157	-0.233
**11**	*Narcisus* cv. Pink Parasol	●	masonine	O=C1C2=CC3=C(OCO3)C=C2C4([H])C(CC=C(CC5)C4([H])N5C)([H])O1	-1.962691	0.961977	0.198064	-0.851	-0.023	1.4159	0.2562	0.0107
**8**	*Narcissus* cv. Dutch Master	●	oduline	OC1C2=CC3=C(OCO3)C=C2C4([H])C(CC=C(CC5)C4([H])N5C)([H])O1	-1.843048	0.682491	-0.10885	-1.146	-0.136	1.2352	-0.183	0.27
**2**	*Zephyranthes robusta*	●	(+)-hamayne	[H]C12N3CC4=CC5=C(OCO5)C=C4C1(C(O)C3)C=CC(O)C2	-1.830899	0.652561	-0.78613	-1.237	-0.146	1.1852	-0.157	0.5292
**9**	*Narcissus cv.* Prof. Einstein	●	lycorine	OC1C=C(CC2)C3([H])N2CC4=CC5=C(OCO5)C=C4C3([H])C1O	-1.830155	0.663384	-0.73599	-1.224	-0.158	1.1786	-0.176	0.5049
**3**	*Zephyranthes robusta*	●	(+)-haemanthamine	OC1C23C(CC(OC)C=C3)N(C1)CC4=CC5=C(OCO5)C=C42	-1.812215	0.605401	-0.2002	-1.012	-0.067	1.0179	-0.115	0.3798
**62**	*Liriodendron tulipifera*	●	liriodenine	O=C1C2=CC=CC=C2C3=C(OCO4)C4=CC5=C3C1=NC=C5	-1.77291	3.985069	0.239336	-0.542	0.2498	1.2953	0.0959	1.0339
**54**	*Papaver somniferum*	●	thebaine	COC1=C2C(C(C(C3C4)=CC=C5OC)(CCN3C)C5O2)=C4C=C1	-1.768508	0.662666	0.689255	-0.764	0.2182	0.9351	0.0654	0.1345
**10**	*Narcisus* cv. Pink Parasol	●	homolycorine	O=C1C2=CC(OC)=C(OC)C=C2C3([H])C(CC=C(CC4)C3([H])N4C)([H])O1	-1.732966	0.644265	0.465713	-0.539	0.1779	0.7391	0.0984	0.0333
**7**	*Narcissus* cv. Dutch Master	●	lycorenine	OC1C2=CC(OC)=C(OC)C=C2C3([H])C(CC=C(CC4)C3([H])N4C)([H])O1	-1.614856	0.378614	0.152226	-0.831	0.0529	0.5876	-0.33	0.2935
**4**	*Zephyranthes robusta*	●	9-*O*-demethylgalanthine	[H]C12C(C(CC3)=CC(OC)C2O)([H])N3CC4=CC(OC)=C(O)C=C41	-1.597896	0.543637	-0.40053	-0.883	0.0707	0.4441	-0.393	0.0012
**13**	*Nerine bowdenii*	●	(-)-Undulatine	COC1CC2N3CC4=C(OC)C5=C(OCO5)C=C4C2(CC3)C6C1O6	-1.588668	0.47971	0.173966	-0.957	-0.315	1.0129	-0.219	0.2977
**12**	*Narcisus* cv. Pink Parasol	●	hippeastrine	O=C1C2=CC3=C(OCO3)C=C2C4([H])C(C(O)C=C(CC5)C4([H])N5C)([H])O1	-1.580716	0.814023	-0.38153	-0.92	-0.165	1.3654	0.3005	0.1899
**5**	*Chlidanthus fragrans*	●	(+)-tazzetine	OC1(CN2C)OCC3=CC4=C(OCO4)C=C3C15C2([H])CC(OC)C=C5	-1.431515	0.481861	-0.18459	-1.015	-0.181	1.0422	-0.088	0.3243
**1**	***Zephyranthes robusta***	●	**(±)-haemanthidine**	OC1C2=CC3=C(OCO3)C=C2C4(C=CC(C5)OC)C5([H])N1CC4O	**-1.418984**	**0.474625**	**-0.71752**	**-1.067**	**-0.222**	**0.958**	**-0.118**	**0.5146**
**49**	*Chelidonium majus*	●	stylopine	[H]C12C3=CC4=C(OCO4)C=C3CCN1CC5=C(C=CC6=C5OCO6)C2	-1.411321	2.418279	0.66138	-1.091	-0.158	1.2783	-0.398	0.5798
**60**	*Peumus boldus*	●	(-)-pallidine	O=C(C1)C(OC)CC2(C1C(N(C)CC2)C3)C4=C3C=C(O)C(OC)=C4	-1.406492	0.465425	0.228917	-0.894	0.1072	0.5554	-0.397	-0.227
**59**	*Peumus boldus*	●	(-)-salutaridine (sinoacutine)	OC1=C(OC)C=CC2=C1C3(C(C(N(C)CC3)C2)=C4)C=C(OC)C4=O	-1.338216	0.756856	0.176906	-0.588	0.2956	0.7188	0.2678	-0.357
**22**	*Berberis vulgaris*	●	8-oxoberberine	C=C1C(C(N(CCC2=C3C=C4C(OCO4)=C2)C3=C1)=O)=C(OC)COC	-1.259559	0.727905	0.28269	-0.109	0.303	0.7481	0.8676	-0.041
**34**	*Eschscholtzia californica*	●	caryachine	OC(C(OC)=C1)=CC2=C1CC3C(C=C(OCO4)C4=C5)=C5CC2N3C	-1.209885	2.351854	0.440033	-0.975	-0.008	0.7049	-0.649	0.2351
**43**	*Fumaria officinalis*	●	(-)-sinactine	OC1=C(OC)C=C2C(C(CC(C=CC3=C4OCO3)=C4C5)N5CC2)=C1	-1.208682	2.350593	0.436035	-0.976	-0.007	0.7058	-0.647	0.2374
**28**	*Corydalis cava*	●	(+)-bulbocapnine	OC1=C(OC)C=CC(C2)=C1C3=C(C2([H])N(C)CC4)C4=CC5=C3OCO5	-1.206864	2.3479	0.426419	-0.979	-0.004	0.7077	-0.643	0.2426
**35**	*Eschscholtzia californica*	●	*O*-methylcaryachine	CN1C2CC3=C(C=C(OCO4)C4=C3)C1CC5=C2C=C(OC)C(OC)=C5	-1.183468	2.103641	0.928653	-0.791	0.0367	0.6191	-0.56	0.5964
**36**	*Eschscholtzia californica*	●	*O*-methylneocaryachine	COC(C=C1)=C(OC)C2=C1CC3C(C=C(OCO4)C4=C5)=C5CC2N3C	-1.183468	2.103641	0.928653	-0.791	0.0367	0.6191	-0.56	0.5964
**26**	*Corydalis cava*	●	(+)-canadine	[H]C12N(CCC3=C2C=C4C(OCO4)=C3)CC5=C(C=CC(OC)=C5OC)C1	-1.182264	2.10238	0.924655	-0.792	0.0379	0.6201	-0.559	0.5987
**33**	*Eschscholtzia californica*	●	(-)-scoulerine	CN1CC2=C(C=CC(OC)=C2O)CC1([H])C3=CC=C(OC)C(O)=C3	-1.159112	2.260837	0.10113	-0.559	0.2898	-0.295	-0.95	-0.054
**57**	*Peumus boldus*	●	norisocorydine	OC1=C2C(CC3C4=C2C(OC)=C(OC)C=C4CCN3)=CC=C1OC	-1.030072	2.124213	0.250835	-0.643	0.2456	0.0309	-0.814	0.2816
**58**	*Peumus boldus*	●	(+)-laurotetanine	OC1=C(OC)C=C2C(CC3([H])C4=C2C(OC)=C(OC)C=C4CCN3)=C1	-1.030072	2.124213	0.250835	-0.643	0.2456	0.0309	-0.814	0.2816
**27**	*Corydalis cava*	●	(+)-thalictricavine	[H]C12C(C)C3=CC=C(OC)C(OC)=C3CN1CCC4=C2C=C(OCO5)C5=C4	-1.004281	2.002902	1.101164	-0.857	0.0328	0.6261	-0.634	0.5513
**24**	*Argemone sp.*	●	platycerine	OC1=C(OC)C=CC2=C1C(N3C)CC4=C(C=C(OC)C(OC)=C4)C3C2	-0.982624	2.05017	0.696528	-0.677	0.1743	0.0809	-0.8	0.254
**25**	*Corydalis cava*	●	(+)-corypalmine	[H]C12C3=CC(OC)=C(O)C=C3CCN1CC4=C(C=CC(OC)=C4OC)C2	-0.981421	2.04891	0.69253	-0.678	0.1755	0.0818	-0.798	0.2563
**32**	*Corydalis yanhusuo*	●	tetrahydrocolumbamine	[H]C12C3=CC(O)=C(OC)C=C3CCN1CC4=C(C=CC(OC)=C4OC)C2	-0.981421	2.04891	0.69253	-0.678	0.1755	0.0818	-0.798	0.2563
**56**	*Peumus boldus*	●	*N*-methyllaurotetanine	[H]C12C3=C(C(C=C(OC)C(O)=C4)=C4C2)C(OC)=C(OC)C=C3CCN1C	-0.979603	2.046217	0.682913	-0.681	0.1782	0.0837	-0.794	0.2615
**41**	*Fumaria officinalis*	●	(+)-fumariline	O=C1C2=C(C=CC3=C2OCO3)CC41C5=CC6=C(OCO6)C=C5CCN4C	-0.974221	2.402647	0.555151	-0.949	-0.177	1.3848	-0.007	0.448
**23**	*Argemone grandiflora*	●	argemonine	CN1C2CC3=C(C=C(OC)C(OC)=C3)C1CC4=C2C=C(OC)C(OC)=C4	-0.95747	1.814994	1.176013	-0.5	0.206	0.0342	-0.706	0.6131
**47**	*Glaucium flavum*	●	glaucine	COC1=C(OC)C(C(C=C(OC)C(OC)=C2)=C2C3)=C(C3N(C)CC4)C4=C1	-0.954449	1.81104	1.162399	-0.504	0.21	0.037	-0.7	0.6207
**53**	*Papaver somniferum*	●	papaverine	COC1=C(OC)C=C(C(CC2=CC=C(OC)C(OC)=C2)=NC=C3)C3=C1	-0.917607	2.84396	1.015875	0.1569	0.5703	-0.724	-0.6	1.1047
**48**	*Glaucium flavum*	●	norchelidonine	OC1CC2=C(C=C(OCO3)C3=C2)C4([H])NCC5=C6C(OCO6)=CC=C5C41[H]	-0.905212	2.224364	-0.22677	-1.214	-0.226	1.2003	-0.421	0.7489
**50**	*Chelidonium majus*	●	chelidonine	OC1CC2=C(C=C(OCO3)C3=C2)C4([H])N(CC5=C6C(OCO6)=CC=C5C41[H])C	-0.852501	2.134509	0.215937	-1.249	-0.273	1.2072	-0.413	0.7348
**31**	*Corydalis yanhusuo*	●	(+)-corydaline	[H]C12N(CCC3=C2C=C(OC)C(OC)=C3)CC4=C(C=CC(OC)=C4OC)C1C	-0.779102	1.723687	1.342547	-0.566	0.1925	0.0639	-0.773	0.5678
**42**	*Fumaria officinalis*	●	(+)-parfumine	OC1=C(OC)C=C2C(C3(CC(C=CC4=C5OCO4)=C5C3=O)N(CC2)C)=C1	-0.772924	2.333913	0.331824	-0.839	-0.028	0.8209	-0.254	0.1025
**29**	*Corydalis cava*	●	allocryptopine	O=C1C2=CC3=C(OCO3)C=C2CCN(C)CC4=C(C=CC(OC)=C4OC)C1	-0.762676	1.986445	0.839723	-0.601	0.1292	0.5486	-0.279	0.4336
**45**	*Fumaria officinalis*	●	cryptopine	O=C1C2=CC(OC)=C(OC)C=C2CCN(C)CC3=C(C=CC4=C3OCO4)C1	-0.762676	1.986445	0.839723	-0.601	0.1292	0.5486	-0.279	0.4336
**46**	*Fumaria officinalis*	●	(+)-bikukuline	O=C(OC1([H])C2([H])C3=CC4=C(OCO4)C=C3CCN2C)C5=C1C=CC6=C5OCO6	-0.742327	2.323148	0.458775	-0.723	-0.267	1.1812	0.0995	0.4532
**61**	*Liriodendron tulipifera*	●	oxoglaucine	COC1=C(OC)C2=C3C(C=CN=C3C(C4=CC(OC)=C(OC)C=C42)=O)=C1	-0.706501	3.101379	0.651493	0.0064	0.3421	0.033	-0.049	0.926
**30**	*Corydalis cava*	●	(+)-canadaline	O=CC1=C(OC)C(OC)=CC=C1CC2C3=C(C=C(OCO4)C4=C3)CCN2C	-0.664186	1.861381	0.997667	-0.145	0.218	-0.08	-0.255	0.5392
**40**	*Fumaria officinalis*	●	(-)-fumaritine	OC1=C(OC)C=C2C(C3(CC(C=CC4=C5OCO4)=C5C3O)N(CC2)C)=C1	-0.641028	2.078823	0.004021	-1.131	-0.133	0.6492	-0.684	0.3677
**38**	*Fumaria officinalis*	●	(-)-fumaricine	OC1C2=C(C=CC3=C2OCO3)CC41C5=CC(OC)=C(OC)C=C5CCN4C	-0.625106	1.840024	0.511264	-0.949	-0.095	0.5809	-0.58	0.7357
**51**	*Chelidonium majus*	●	6-ethoxydihydrosanguinarine	CN1C(OCC)C2=C(C=CC3=C2OCO3)C4=C1C5=CC(OCO6)=C6C=C5C=C4	-0.368389	3.373787	1.130925	-0.773	-0.075	0.7011	-0.483	0.8475
**44**	*Fumaria officinalis*	●	(±)-*O*-methylfumarofine	OC12C(C3=C(C1N(CCC4=CC(OC)=C(C=C24)OC)C)C=CC5=C3OCO5)=O	-0.367352	1.930486	0.255195	-0.749	-0.112	0.6882	-0.126	0.6394
**37**	*Fumaria officinalis*	●	(-)-fumarofycine hydrochloride	OC1=C(OC)C=C2C(C3(CC(C=CC4=C5OCO4)=C5C3OC(C)=O)N(CC2)C)=C1	-0.157681	1.990851	0.550093	-0.646	-0.057	0.4346	-0.258	0.1015
**52**	*Chelidonium majus*	●	6-ethoxydihydrochelerythrine	CN1CC2=C(C=CC(OC)=C2OC)C3=C1C4=CC(OCO5)=C5C=C4C=C3OCC	-0.140285	3.072246	1.371582	-0.505	0.1069	0.1133	-0.634	0.8674
**39**	*Fumaria officinalis*	●	(-)-*O*-methylfumarofycine	CN1C2(CC(C=CC3=C4OCO3)=C4C2OC(C)=O)C5=CC(OC)=C(OC)C=C5CC1	-0.13169	1.760397	1.029333	-0.481	-0.018	0.3909	-0.171	0.4645
**55**	*Papaver somniferum*	●	(-)-narcotine	O=C(C1=C2C=CC(OC)=C1OC)OC2([H])C3([H])C4=C(OC)C5=C(OCO5)C=C4CCN3C	-0.104135	1.8313	0.78075	-0.342	-0.142	0.3912	-0.028	0.4452
**20**	*Berberis vulgaris*	●	(-)-muraricine	OC(C=C1)=CC=C1CC2C3=CC(OC4=C(OC)C(OC)=CC(CC5)=C4CN5C)=C(OC)C=C3CCN2C	1.594389	2.623085	2.130938	-0.518	0.2621	-0.199	-1.177	0.4891
**16**	***Berberis vulgaris***	●	**(+)-aromoline**	OC(C(O1)=C2)=CC=C2CC(C3=C(CC4)C=C(OC)C(OC5=C(C(CC6=CC=C1C=C6)([H])N(CC7)C)C7=CC(OC)=C5O)=C3)([H])N4C	**3.180525**	**4.065469**	**2.445422**	**-1.467**	**-0.082**	**0.7009**	**-1.461**	**0.1158**
**19**	***Berberis vulgaris***	●	**(+)-obamegine**	OC1=C(OC2=C(OC)C=C3C(C(CC4=CC=C5C=C4)([H])N(CC3)C)=C2)C6=C(CCN(C6([H])CC7=CC=C(O)C(O5)=C7)C)C=C1OC	**3.180525**	**4.065469**	**2.445422**	**-1.467**	**-0.082**	**0.7009**	**-1.461**	**0.1158**
**17**	***Berberis vulgaris***	●	**(+)-berbamine**	OC(C(O1)=C2)=CC=C2CC(C3=C(CC4)C=C(OC)C(OC)=C3OC5=C(OC)C=C6C(C(CC7=CC=C1C=C7)([H])N(CC6)C)=C5)([H])N4C	**3.199309**	**3.855098**	**2.921477**	**-1.302**	**-0.069**	**0.7065**	**-1.357**	**0.4809**
**18**	***Berberis vulgaris***	●	**(+)-bersavine**	OC(C(O1)=C2)=C(CN(CC)CC)C=C2CC(C3=C(CC4)C=C(OC)C(OC)=C3OC5=C(OC)C=C6C(C(CC7=CC=C1C=C7)([H])N(CC6)C)=C5)([H])N4C	**4.419066**	**3.258206**	**3.703242**	**-0.991**	**0.0788**	**0.428**	**-1.64**	**0.5311**
**21**	*Berberis vulgaris*	●	(-)-berkristine	O=C1C2=C(C=C(OC)C(OC3=C(C(CC4=CC=C(OC5=CC(C6NC(C)(C)CC(C6)=O)=CC=C5O)C=C4)N(CC7)C)C7=CC(OC)=C3OC)=C2)CCN1C	5.723366	3.046751	3.082615	-0.432	-0.224	0.0849	-0.549	1.1257

Alkaloids are listed by increasing PC1 (➔) score in the table (please refer to the **red axis** in Figure 4)

Table S3. ChemGPS-NP data of in-house database for anti-coronavirus drugs (available in Excel format)

Clinical, preclinical or experimental anti-coronavirus drugs including chemical antivirotics (●), indole derivatives (●), and pink natural alkaloid antivirotics (●)		ChemGPS-NP data									
Compound name	*SMILES (Pubchem)*	PC1 (→)	PC2 (→)	PC3 (→)	PS4	PS5	PS6	PS7	PS8	References	
**Clinical and preclinical chemical antivirotics (●)**											
favipiravir	C1=C(N=C(C(=O)N1)C(=O)N)F	-3.67872	-0.02819	-2.945334	1.5084	0.2115	1.148	1.0075	-0.546	Bezmialem Sci., 2020, 8 (Suppl. 3), 131-9	
chlorpromazine	CN(CCCN1C2=CC=CC=C2SC3=CC=C(Cl)C=C31)C	-1.957745	2.283394	1.290008	0.1156	0.6424	-0.009	-1.057	0.8014	Bezmialem Sci., 2020, 8 (Suppl. 3), 131-9	
ribavirin	OCC1C(C(C(N2C=NC(C(N)=O)=N2)O1)O)O	-1.876967	-0.17637	-3.504411	0.4248	0.6095	0.0544	-0.165	0.8599	Bezmialem Sci., 2020, 8 (Suppl. 3), 131-9	
chloroquine	CC(CCCN(CC)CC)NC1=CC=NC2=CC(Cl)=CC=C12	-1.6275	1.14143	1.261906	0.7137	1.0922	-0.879	-1.303	0.8248	Bezmialem Sci., 2020, 8 (Suppl. 3), 131-9	
hydroxychloroquine	CCN(CCCC(NC1=C2C=CC(Cl)=CC2=NC=C1)C)CCO	-1.204448	0.929682	0.695615	0.7699	0.9883	-1.119	-1.253	1.0277	Bezmialem Sci., 2020, 8 (Suppl. 3), 131-9	
loperamide	CN(C(C(C1=CC=CC=C1)(C2=CC=CC=C2)CCN3CCC(CC3)(C4=CC=C(C=C4)Cl)O)=O)C	0.931983	2.639004	2.108493	-0.188	0.5559	-0.186	-1.099	1.078	Bezmialem Sci., 2020, 8 (Suppl. 3), 131-9	
**remdesivir**	**CCC(COC(=O)C(NP(=O)(Oc1ccccc1)OCC1OC(C(C1O)O)(C#N)c1ccc2n1ncnc2N)C)CC**	**3.913509**	**0.792135**	**-0.1455**	**1.2124**	**0.7136**	**-0.747**	**-0.03**	**1.7436**	**Bezmialem Sci., 2020, 8 (Suppl. 3), 131-9**	
**lopinavir**	**CC(C(C(NC(CC1=CC=CC=C1)CC(C(NC(COC2=C(C=CC=C2C)C)=O)CC3=CC=CC=C3)O)=O)N4CCCNC4=O)C**	**3.977594**	**1.594707**	**2.210516**	**0.4995**	**1.1439**	**-0.825**	**-0.954**	**0.7759**	**Bezmialem Sci., 2020, 8 (Suppl. 3), 131-9**	
ritonavir	CC(C)C(C(NC(CC1=CC=CC=C1)CC(O)C(CC2=CC=CC=C2)NC(OCC3=CN=CS3)=O)=O)NC(N(C)CC4=CSC(C(C)C)=N4)=O	5.412066	1.551184	2.116008	1.3011	1.0648	-0.875	-0.478	1.7044	Bezmialem Sci., 2020, 8 (Suppl. 3), 131-9	
**Indole synthetic antivirotics (●)**											
**11p** (CHEMBL4474046)	C#CCC(NC(=O)C=Cc1ccccc1)C(=O)NC(CC1CCNC1=O)C(=O)C(=O)NCc1ccccc1	2.206268	1.217047	0.723323	1.2392	1.5794	-0.517	0.5052	0.1715	J. Med. Chem., 2020, 63, 4562-4578	
**11n** (CHEMBL4590737)	CC(C)CC(NC(=O)C=Cc1ccccc1)C(=O)NC(CC1CCNC1=O)C(=O)C(=O)NCc1ccccc1	2.492729	0.890141	0.966029	1.1128	1.5956	-0.613	0.3011	0.1463	J. Med. Chem., 2020, 63, 4562-4578	
**11s** (CHEMBL4442435)	O=C(C=Cc1ccccc1)NC(CC1CC1)C(=O)NC(CC1CCNC1=O)C(=O)C(=O)NCc1ccccc1	2.510692	0.965927	0.898235	1.0225	1.5238	-0.456	0.4133	0.2128	J. Med. Chem., 2020, 63, 4562-4578	
**11o** (CHEMBL4465975)	CCCCC(NC(=O)C=Cc1ccccc1)C(=O)NC(CC1CCNC1=O)C(=O)C(=O)NCc1ccccc1	2.523104	0.860057	1.033764	1.2382	1.6124	-0.778	0.2975	0.1652	J. Med. Chem., 2020, 63, 4562-4578	
**11t** (CHEMBL4542462)	O=C(C=Cc1ccccc1)NC(CC1CCC1)C(=O)NC(CC1CCNC1=O)C(=O)C(=O)NCc1ccccc1	2.687284	0.900491	1.08708	0.9575	1.498	-0.404	0.3359	0.1631	J. Med. Chem., 2020, 63, 4562-4578	
**11u** (CHEMBL4438019)	O=C(C=Cc1ccccc1)NC(CC1CCCC1)C(=O)NC(CC1CCNC1=O)C(=O)C(=O)NCc1ccccc1	2.864141	0.836371	1.275101	0.897	1.4714	-0.354	0.2599	0.1146	J. Med. Chem., 2020, 63, 4562-4578	
**11r** (CHEMBL4441971)	O=C(C=Cc1ccccc1)NC(CC1CCCCC1)C(=O)NC(CC1CCNC1=O)C(=O)C(=O)NCc1ccccc1	3.041234	0.773443	1.462383	0.8406	1.4442	-0.306	0.1852	0.0672	J. Med. Chem., 2020, 63, 4562-4578	
**11a (CHEMBL3780681)**	O=C(C=Cc1ccccc1)NC(Cc1ccccc1)C(=O)NC(CC1CCNC1=O)C(=O)C(=O)NCc1ccccc1	**3.266018**	**2.255681**	**1.30836**	**0.9783**	**1.617**	**-0.552**	**0.234**	**0.4622**	**J. Med. Chem., 2020, 63, 4562-4578**	
**11m** (CHEMBL4572853)	O=C(C=Cc1ccccc1)NC(Cc1ccc(F)cc1)C(=O)NC(CC1CCNC1=O)C(=O)C(=O)NCc1ccccc1	3.35592	2.283491	1.290083	1.2496	1.3492	-0.423	0.1643	0.4187	J. Med. Chem., 2020, 63, 4562-4578	
**Natural alkaloid antivirotics (●)**											
tryptanthrin	C1=CC=C2C(=C1)C(=O)N3C4=CC=CC=C4C(=O)C3=N2	-2.190603	3.229331	-0.059461	-0.168	0.6191	1.4819	0.5531	0.6123	Bezmialem Sci., 2020, 8 (Suppl. 3), 131-9	
**lycorine**	OC1C=C(CC2)C3([H])N2CC4=CC5=C(OCO5)C=C4C3([H])C1O	-1.830155	0.663384	-0.735985	-1.224	-0.158	1.1786	-0.176	0.5049	Bezmialem Sci., 2020, 8 (Suppl. 3), 131-9	
indirubin	O=C(C1=C2NC3=C(C2=O)C=CC=C3)NC4=C1C=CC=C4	-1.759228	2.995284	-0.3763	-0.366	0.7858	1.3475	0.2687	0.5114	Bezmialem Sci., 2020, 8 (Suppl. 3), 131-9	
indigo	O=C(C(N1)=C2NC(C=CC=C3)=C3C2=O)C4=C1C=CC=C4	-1.746638	3.001349	-0.330805	-0.357	0.7778	1.3455	0.2529	0.4928	Bezmialem Sci., 2020, 8 (Suppl. 3), 131-9	
indigotin	O=C1C(NC2=C1C=CC=C2)=C3NC4=C(C3=O)C=CC=C4	-1.746638	3.001349	-0.330805	-0.357	0.7778	1.3455	0.2529	0.4928	Bezmialem Sci., 2020, 8 (Suppl. 3), 131-9	
indigodole B	O=C1C(N2C3=C1C=CC=C3)(CC)NC4=CC=CC=C4C2=O	-1.731688	2.666446	0.284981	-0.393	0.7135	0.9034	-0.116	0.5715	Bezmialem Sci., 2020, 8 (Suppl. 3), 131-9	
quinine	[H]C1(C(C2=CC=NC3=CC=C(C=C23)OC)O)CC4CCN1CC4C=C	-1.20705	1.253828	0.713873	-0.465	0.6798	0.1934	-0.551	0.8001	Bezmialem Sci., 2020, 8 (Suppl. 3), 131-9	
moupinamide	COC1=C(C=CC(=C1)C=CC(=O)NCCC2=CC=C(C=C2)O)O	-0.859813	2.267869	-0.125705	0.3098	0.7515	-1.097	-0.453	-0.126	Bezmialem Sci., 2020, 8 (Suppl. 3), 131-9	
tylophorine	COC1=C(C=C2C(=C1)C3=C(CN4CCCC4C3)C5=CC(=C(C=C52)OC)OC)OC	-0.178164	2.678866	1.633795	-0.718	0.2194	0.1282	-0.809	0.8149	Bezmialem Sci., 2020, 8 (Suppl. 3), 131-9	
emetine	CCC1C(CC2NCCC3=C2C=C(OC)C(OC)=C3)CC4C5=C(C=C(C(OC)=C5)OC)CCN4C1	0.898285	1.029683	2.174189	-0.587	0.2609	0.0908	-1.17	0.5061	Bezmialem Sci., 2020, 8 (Suppl. 3), 131-9	
10-hydroxyusambarensine	CC=C1CN2CCC3=C(C2CC1CC4=NC=CC5=C4NC6=C5C=C(C=C6)O)NC7=CC=CC=C37	1.34112	3.973809	1.77397	-0.989	1.1352	1.0924	-1.253	0.7529	Bezmialem Sci., 2020, 8 (Suppl. 3), 131-9	
mycalamide A	CC1C(OC(CC1=C)(C(C(=O)NC2C3C(C(C(C(O3)CC(CO)O)(C)C)OC)OCO2)O)OC)C	1.519638	-2.54782	-0.871325	-0.363	-0.886	-0.288	0.2086	0.7845	RSC Adv., 2020, 10, 23379–23393	
cryptoquindoline	CN(C1=C2C=CC=C1)C3=C4C(C=CC=C4)=NC3=C2N5C6=CC7=CC=CC=C7N=C6C8=C5C=CC=C8	1.578064	7.305794	3.01624	-0.954	1.1956	1.2315	-1.355	1.9586	Bezmialem Sci., 2020, 8 (Suppl. 3), 131-9	
sabadinine	CC1CCC2C(C3(C(CC4(C5CCC6C7(C5(CC4(C3CN2C1)O)OC6(C(CC7)O)O)C)O)O)O)(C)O	1.97718	-2.98742	-1.961001	-2.66	-1.727	1.2887	-1.038	1.4766	Bezmialem Sci., 2020, 8 (Suppl. 3), 131-9	
desmethoxyreserpine	COC1C(CC2CN3CCC4=C(C3CC2C1C(=O)OC)NC5=CC=CC=C45)OC(=O)C6=CC(=C(C(=C6)OC)OC)OC	2.528893	1.582729	1.971795	-0.186	0.197	0.1153	-0.174	0.5032	Bezmialem Sci., 2020, 8 (Suppl. 3), 131-9	
**cepharanthine**	CN1CCC2=C(C(OC3=CC(C4([H])N(C)CC5)=C5C=C3OC)=C(OCO6)C6=C2)C1([H])CC7=CC=C(OC8=C(OC)C=CC(C4)=C8)C=C7	**3.007471**	**3.883223**	**3.161811**	**-1.399**	**-0.17**	**1.1462**	**-1.144**	**0.8332**	Bezmialem Sci., 2020, 8 (Suppl. 3), 131-9	
**fangchinoline (tetrandrine)**	CN1C(CC2=CC=C(C(OC(C=C3)=CC=C3C4)=C2)OC)C(C(OC5=C(OC)C=C6CCN(C)C4C6=C5)=C(OC)C(OC)=C7)=C7CC1	**3.227014**	**3.646123**	**3.376822**	**-1.141**	**-0.053**	**0.716**	**-1.267**	**0.8418**	Bezmialem Sci., 2020, 8 (Suppl. 3), 131-9	
**Natural terpenes (dark green dots, ●)**											
dehydroabieta-7-one	CC(C)C1=CC2=C(C=C1)C3(CCCC(C3CC2=O)(C)C)C	-2.275411	0.479051	1.597422	-1.132	0.2966	0.4444	-0.844	-0.147	J. Med. Chem., 2007, 50, 17	*diterpene*
8-beta-hydroxyabiet-9(11),13-dien-12-one	O=C1C(C(C)C)=CC2(O)CCC3C(C)(C)CCCC3(C)C2=C1	-2.105606	-1.26215	1.003197	-1.115	0.1144	0.7502	-0.222	-0.668	RSC Adv., 2020, 10, 23379–23393	*diterpene*
pinusolidic acid	CC12CCCC(C)(C(O)=O)C1CCC(C2CCC3=CCOC3=O)=C	-1.541752	-1.24832	0.834136	-0.392	0.0915	0.2321	0.3338	-0.677	J. Med. Chem., 2007, 50, 17	*diterpene*
7-beta-hydroxydeoxycryptojaponol	CC(C)C1=C(OC)C(O)=C(C2(C)C(CC3O)C(C)(C)CCC2)C3=C1	-1.349939	0.130258	0.88737	-1.262	-0.086	0.0447	-1.287	-0.313	RSC Adv., 2020, 10, 23379–23393	*diterpene*
3-beta,12-diacetoxyabiet-6,8,11,13-tetraene	CC(C)C1=C(C(C)=O)C(C)=C(C2(C)C(CC3)C(C)(C)C(C(C)=O)CC2)C3=C1	-1.052387	0.09381	2.100041	-0.878	0.2806	0.2818	-0.614	-0.27	RSC Adv., 2020, 10, 23379–23393	*diterpene*
iguesterin	CC1=CCC2(CCC3(C4=CC=C5C(=C(C(=O)C=C5C4(CCC3(C2C1)C)C)O)C)C)C	-0.561748	-1.1301	2.021315	-1.314	0.1704	1.5971	0.465	-0.9	RSC Adv., 2020, 10, 23379–23393	*steroid triterpene*
tingenone	CC1(C2=CC=C(C(C)=C3O)C4=CC3=O)C(CCC24C)(C)C(CC5C)([H])C(CC5=O)(C)CC1	-0.335189	-1.1825	1.690519	-1.301	0.1143	1.6195	0.6316	-0.852	RSC Adv., 2020, 10, 23379–23393	*steroid triterpene*
forskolin	CC(=O)OC1C(C2C(C(CCC2(C)C)O)(C3(C1(OC(CC3=O)(C)C=C)C)O)C)O	-0.28464	-1.85726	-0.57136	-1.018	-0.313	0.3968	0.138	-0.151	J. Med. Chem., 2007, 50, 17	*diterpene*
betunolic acid (=betulonic)	O=C(C(CCC1(C)C2(C)CCC3([H])C4(C)C)(CCC5C(C)=C)C5([H])C1([H])CCC2([H])C3(C)CCC4=O)O	-0.025034	-1.72235	2.31837	-1.425	-0.274	1.1226	-0.497	-0.715	RSC Adv., 2020, 10, 23379–23393	*steroid triterpene*
pristimerin	O=C1C(O)=C(C)C2=CC=C(C(CCC3(C)C4([H])CC(C(OC)=O)(C)CC3)(C)C4(C)CC5)C5(C)C2=C1	0.285397	-1.38404	2.030134	-1.054	0.019	1.3183	0.5794	-0.876	RSC Adv., 2020, 10, 23379–23393	*steroid triterpene*
**Other natural compounds (grey dots, ●)**											
caffeic acid	OC1=CC(C=CC(O)=O)=CC=C1O	-2.967385	1.626564	-1.83297	0.2874	0.182	-0.572	0.0131	-0.277	Virus Res., 2020, 284, 197989	*phenolic*
isolinoleic acid	CC(C)CCC=CCC=CCCCCCCCC(O)=O	-2.120245	-1.92187	1.589526	1.132	0.7111	-2.412	-0.358	-0.454	RSC Adv., 2020, 10, 23379–23393	*fatty acid*
emodin	CC1=CC2=C(C(=C1)O)C(=O)C3=C(C2=O)C=C(C=C3O)O	-1.482951	3.291749	-1.13634	-0.577	-0.033	0.4995	-0.052	-0.643	Antiviral Research, 2005, 68(1), 36-42	*phenolic*
hesperitine (hesperetine)	COC1=C(C=C(C=C1)C2CC(=O)C3=C(C=C(C=C3O2)O)O)O	-1.094547	2.799805	-0.94964	-0.432	-0.088	-0.201	-0.359	-0.548	Antiviral Research, 2005, 68(1), 36-42	*flavonoid*
indican	C1=CC=C2C(=C1)C(=CN2)OC3C(C(C(C(O3)CO)O)O)O	-1.056445	0.974185	-2.16818	-0.617	0.0351	-0.329	-0.663	1.2509	Bezmialem Sci., 2020, 8 (Suppl. 3), 131-9	*glycosid alkaloid*
scutellarein	O=C(C1=C(O)C(O)=C(O)C=C1O2)C=C2C3=CC=C(O)C=C3	-1.049053	3.264048	-1.59606	-0.393	-0.123	0.0945	-0.1	-0.986	RSC Adv., 2020, 10, 23379–23393	*flavonoid*
savinin	O=C1OCC(CC(C=C2)=CC3=C2OCO3)C1=CC(C=C4)=CC5=C4OCO5	-0.88139	2.36406	0.666553	-0.268	-0.169	0.4798	0.3706	0.3618	RSC Adv., 2020, 10, 23379–23393	*lignan*
sinigrin	C=CCC(SC1C(O)C(O)C(O)C(CO)O1)=NOS(=O)(O)=O	-0.699991	-1.65442	-3.22766	0.88	-0.131	-1.111	0.9627	0.3024	Bezmialem Sci., 2020, 8 (Suppl. 3), 131-9	*glucosinolate*
isobavachalcone	OC1=C(C(C=CC2=CC=C(O)C=C2)=O)C=CC(O)=C1CC=C(C)C	-0.654582	2.463633	0.322687	-0.012	0.5507	-0.995	-0.374	-0.793	Virus Res., 2020, 284, 197989	*chalcone*
quercetin	OC1=C(C2=CC=C(O)C(O)=C2)OC3=C(C(O)=CC(O)=C3)C1=O	-0.639214	3.099599	-2.1874	-0.455	-0.279	0.0383	-0.106	-0.861	Virus Res., 2020, 284, 197989	*flavonoid*
herbacetin	C1=CC(=CC=C1C2=C(C(=O)C3=C(O2)C(=C(C=C3O)O)O)O)O	-0.639214	3.099599	-2.1874	-0.455	-0.279	0.0383	-0.106	-0.861	Virus Res., 2020, 284, 197989	*flavonoid*
myricetin	OC1=C(C2=CC(O)=C(O)C(O)=C2)OC3=C(C(O)=CC(O)=C3)C1=O	-0.223089	3.138994	-2.69222	-0.49	-0.407	-0.076	-0.188	-1.274	RSC Adv., 2020, 10, 23379–23393	*flavonoid*
xanthoangelol E	CC(=C)C(CC1=C(C=CC(=C1O)C(=O)C=CC2=CC=C(C=C2)O)OC)OO	0.003809	1.941349	0.233846	0.4316	0.3699	-1.473	-0.134	-0.261	RSC Adv., 2020, 10, 23379–23393	*chalcone*
silvestrol	COC1C(OC(CO1)C(CO)O)OC2=CC3=C(C(=C2)OC)C4(C(C(C(C4(O3)C5=CC=C(C=C5)OC)C6=CC=CC=C6)C(=O)OC)O)O	4.649688	1.527173	0.320432	-0.618	-1.218	-0.885	0.1003	2.0456	RSC Adv., 2020, 10, 23379–23393	*flavagline*
tannic acid	O=C(C1=CC(O)=C(C(O)=C1)O)OC2C(O)C(OC(COC(C3=CC(O)=C(C(O)=C3)O)=O)C2O)OC(C4=CC(O)=C(O)C(O)=C4)=O	5.932101	3.055839	-3.52528	-0.198	-1.292	-2.248	-0.069	-1.942	RSC Adv., 2020, 10, 23379–23393	*tannin, phenolic*
saikosaponin B2	CC1C(C(C(C(O1)OC2CCC3(C(C2(C)CO)CCC4(C3C=CC5=C6CC(CCC6(C(CC54C)O)CO)(C)C)C)C)O)OC7C(C(C(C(O7)CO)O)O)O)O	6.187546	-3.89598	-0.75856	-2.415	-1.995	0.4518	-0.694	1.6774	Virus Res., 2020, 284, 197989	*steroid saponin*
glycyrrhizin	CC1(C)C(OC2C(OC3C(O)C(O)C(O)C(C(O)=O)O3)C(O)C(O)C(C(O)=O)O2)CCC4(C)C1([H])CCC(C(CCC5(C)C6([H])CC(C(O)=O)(C)CC5)(C)C6=C7)(C)C4([H])C7=O	6.817911	-3.40871	-0.62336	-1.794	-1.844	0.5934	0.5942	1.1078	RSC Adv., 2020, 10, 23379–23393	*steroid saponin*
3-isotheaflavin-3-gallate	C1C(C(OC2=CC(=CC(=C21)O)O)C3=CC4=C(C(=C(C=C4C5C(CC6=C(C=C(C=C6O5)O)O)OC(=O)C7=CC(=C(C(=C7)O)O)O)O)O)O)C(=O)C(=C3)O)O	7.250637	4.39988	-2.00951	-1.426	-1.326	-0.913	-0.326	-1.999	RSC Adv., 2020, 10, 23379–23393	*phenolic*
dieckol	C(C=C(C7=C6OC8=C(C=C(C=C8O7)O)O)O)O)O)O)O)O)O	8.091928	7.43168	-1.68478	-1.39	-1.788	-1.644	-1.426	-2.324	RSC Adv., 2020, 10, 23379–23393	*phenolic*

The drugs in respective subgroups are listed by increasing PC1 (→) score in the table (please refer to the **red axis** in Figure 4)

The anti-coronavirus reference drugs in-house databse was established accrding to the several REVIEW articles (Virus Research, 2020, 284, 197989; RSC Advance, 2020, 10, 23379–23393; Bezmialem Science, 2020, 8 (Suppl. 3), 131-9; Antiviral Research, 2005, 68(1), 36-42; Journal of Medicinal Chemistry, 2007, 50, 17; Journal of Medicinal Chemistry, 2020, 63, 4562-4578) and ChEMBL list of compounds with the keyword "coronavirus" at https://www.ebi.ac.uk/chembl/.

Materials and Methods

Table S4. Compounds and their sources.

**Plant**	**Plant part**	**Isolated alkaloid**	**Literature**
Zephyranthes robusta Baker	bulbs	(±)-haemanthidine (**1**)<br>(+)-hamayne (**2**)<br>(+)-haemanthamine (**3**)<br>9-*O*-demethylgalanthine (**4**)	1
Chlidanthus fragrans Herb	bulbs	(+)-tazettine (**5**)	2
Narcissus pseudonarcissus L cv. Dutch Master	bulbs	seco-isopowellaminone (**6**)<br>lycorenine (**7**)<br>oduline (**8**)	3
Narcissus cv. Professor Einstein	bulbs	lycorine (**9**)	4
Narcissus poeticus L. cv. Pink Parasol	bulbs	homolycorine (**10**)<br>masonine (**11**)<br>hippeastrine (**12**)	5
Nerine bowdenii W.Watson	bulbs	(–)-undulatine (**13**)<br>(–)-crinine (**14**)<br>(–)-caranine (**15**)	6
Berberis vulgaris L.	root bark	(+)-aromoline (**16**)<br>(+)-berbamine (**17**)<br>(+)-bersavine (**18**)<br>(+)-obamegine (**19**)<br>(–)-muraricine (**20**)<br>(–)-berkristine (**21**)<br>8-oxoberberine (**22**)	7
Argemone platyceras Link & Otto	aerial parts	(–)-platycerine (**24**)	8
Corydalis cava Schweigg. & Körte	tubers	(–)-corypalmine (**25**)<br>(+)-canadine (**26**)<br>(+)-thalictricavine (**27**)<br>(+)-bulbocapnine (**28**)<br>allocryptopine (**29**)<br>(+)-canadaline (**30**)<br>(+)-corydaline (**31**)	9
Corydalis yanhusuo W.T.Wang ex Z.Y.Su & C.Y.Wu	tubers	tetrahydrocolumbamine (**32**)	10
Eschscholtzia californica Cham.	aerial parts and roots	argemonine (**23**)<br>(–)-scoulerine (**33**)<br>(–)-caryachine (**34**)<br>(–)-*O*-methylcaryachine (**35**)<br>*O*-methylneocarychine (**36**)	11
Fumaria officinalis Chaub.	whole dried plant	(–)-fumarophycine hydrochloride (**37**)<br>(–)-fumaricine (**38**)<br>(–)-*O*-methylfumarophycine (**39**)	12

Plant	Plant part	Isolated alkaloid	Literature
		(–)-fumaritine (**40**)<br>(+)-fumariline (**41**)<br>(+)-parfumine (**42**)<br>(–)-sinactine (**43**)<br>(±)-*O*-methylfumarofine (**44**)<br>cryptopine (**45**)<br>(+)-bicuculline (**46**)	
Glaucium flavum Crantz	aerial parts	norchelidonine (**48**)	[13]
Chelidonium majus L.	aerial parts	(–)-stylopine (**49**)<br>chelidonine (**50**)<br>6-ethoxydihydrosanguinarine (**51**)<br>6-ethoxydihydrochelerytrine (**52**)	[14]
Papaver somniferum L.	aerial parts	papaverine (**53**)<br>thebaine (**54**)<br>(–)-narcotine (**55**)	[15-17]
Peumus boldus Molina	leaves	*N*-methyllaurotetanine (**56**)<br>norisocoridine (**57**)<br>(+)-laurotetanine (**58**)<br>(–)-salutaridine (**59**)<br>(–)-pallidine (**60**)	[18]
Liriodendron tulipifera L.	wood	glaucine (**47**)<br>oxoglaucine (**61**)<br>liriodenine (**62**)	[19,20]

Detailed plant extractions, the isolation of pure compounds, NMR spectra, and HRMS analysis are defined in previous reports. The purity of all isolated alkaloids (>95%) was confirmed by NMR spectroscopy and GC/MS analysis.

2.1 Coronavirus 229E assay

The protective effects of the samples against human coronavirus (HCoV) 229E were assessed using a method similar to the previously described one [21,22]. Huh7 cells (human liver carcinoma cell line) were infected with 9TCID50 (Median Tissue Culture Infectious Dose) of each coronavirus 229E with or without the compounds or vehicle. After six days of incubation at 33°C, surviving cells were stained with MTT, and the survival percentage was calculated.

2.2 Pseudotyped lentivirus assay

Stable hACE-2 overexpressed HEK293T cells were provided by Dr. Rei-Lin Kuo (Chang Gung University) and maintained in DMEM containing 10 % FBS and 10 μg/mL blasticidin. VSV-G pseudotyped lentivirus control (clone name: S3w.Fluc.Ppuro) and SARS-CoV-2 S-protein expressing VSV-G pseudotyped lentiviruses (clone name: nCoV-S-Luc-D614G and nCoV-S-Luc-B.1.617.2) were purchased from RNAi Core Facility of Academia Sinica. hACE-2 overexpressed

cells (1×10^4 cells/well) were seeded on 96-well plates and incubated at 37 °C with 5% CO_2. Equal relative infection units (RIU) (5×10^3 RIU/well= 0.5 RIU/cell) of pseudotyped lentiviruses were pretreated with various concentrations of antiviral agents in DMEM containing 5% FBS at 37 °C for 1 hour. The medium of ACE-2 overexpressed cells was replaced with treated pseudotyped lentivirus and cultured for 24 h. Luciferase activity was measured using a Luciferase Assay System (E2520, Promega) and recorded by a Fluorescence Reader.

2.3 WST-1 viability assay

The potential cytotoxicity of tested samples was evaluated by WST-1 reduction assay in hACE-2-overexpressed HEK293T [23]. The hACE-2- overexpressed HEK293T cells (1×10^4 cells/well) were preincubated with DMSO or tested agents for 24 h. Then, the WST-1 reagent (M192427, Sigma-Aldrich, MO, USA) was added and incubated for 4 h at 37°C. The absorbance at 405 nm was measured by a Multiska GO spectrophotometer (Thermo Fisher Scientific, MA, USA).

2.4 Molecular docking

To evaluate the binding energy between **16** and SARS-CoV-2 S-protein in the binding site with ACE2 receptor, molecular docking was performed using Autodock 4.2 with a Lamarckian genetic algorithm [24]. The S-protein structure (PDB ID: 6M0J) was obtained from the Protein Data Bank (http://www.rcsb.org). Cocrystallized protein substrates, ligands, and water were removed, and the polar hydrogens and Kollman United Atom charges were added into the structure of HNE protein using AutoDock Tool 1.5.4 software. The structures of **16**, cepharantine, and S-protein inhibitor nafamostat were optimized utilizing MMFF94 force-field energy minimization in ChemBio3D (version 16.0; PerkinElmer, Waltham, MA, USA). Hydrogens and Gasteiger charges were added to the structure of these ligands by the AutoDock Tool. The S-protein binding location defined the docking site to the ACE2 receptor (X:39.839390; Y:31.431017; Z:6.225943). The grid box was calculated by AutoGrid at the center of the inhibitor molecule binding with dimensions 40×40×40 grid points with a spacing of 0.375 Å. Docking parameters were set as defaults except for the maximum number of energy evaluations, which was increased to 25,000,000 per run. The interaction energy was calculated to analyze the binding between **16**, cepharantine or S-protein inhibitor nafamostat and S-protein by using AutoDock Tool software. The best ligand-binding pose was chosen after evaluating the top 10 binding positions. The docking results were further analyzed and presented using Discovery Studio Visualizer version 4.0 (BIOVIA, San Diego, CA, USA).

2.5 ChemGPS-NP analysis

The ChemGPS-NP principal component analysis of alkaloids and reference anti-coronavirus drugs was calculated using the online tool ChemGPS-NPWeb (http://chemgps.bmc.uu.se) based on

SMILES input from ChemBioDraw (version 17.0) or PubChem (https://pubchem.ncbi.nlm.nih.gov) [25]. Three out of eight dimensions were plotted using the software Grapher (version 2.7, MacOS). These three axes represent size, shape, and polarizability (PC1, x, red axis); aromaticity and conjugation-related properties (PC2, y, blue axis); and lipophilicity, polarity, and hydrogen bond capacity (PC3, z, green axis). The alkaloids were marked as anti-coronavirus 229E (red dots; **1**, **16**, **17**, **18**, **19**) or inactive (blue dots). The reference drugs were classified into several groups, including chemical antivirotics (yellow dots) [26], indole derivatives (green dots) [27], and natural alkaloid antivirotics (pink dots) [26,28]. These are listed in Supporting Information by increasing the PC1 score.

2.6 Statistical analysis

Results are expressed as the value of the mean of two independent measurements (EC_{50} calculation for coronavirus HCoV-229E assay) after a single dose screening of 62 alkaloids (coronavirus HCoV-229E assay, single measurement), and as means ± S.E.M. of three independent measurements (pseudovirus neutralization assay). Comparisons were carried out using Student's *t*-test (SigmaPlot, Jandel Scientific, San Rafael, CA, USA). Statistical significance was acceptable at a level of $p < 0.05$. The 50% inhibitory concentration (IC_{50}) or 50% effective concentration (EC_{50}) were calculated from the dose-response curve obtained by plotting the percentage of inhibition versus concentration (linear function, Microsoft Office). Statistical significance was acceptable at a level of $p < 0.05$.

References

1. Kulhankova A, Cahlikova L, Novak Z, Macakova K, Kunes J, Opletal L. Alkaloids from *Zephyranthes robusta* BAKER and their acetylcholinesterase- and butyrylcholinesterase-inhibitory activity. *Chem Biodivers*. Jun 2013;10(6):1120-7. doi:10.1002/cbdv.201200144
2. Cahlíková L, Hrabinová M, Kulhánková A, et al. Alkaloids from *Chlidanthus fragrans* and their acetylcholinesterase, butyrylcholinesterase and prolyl oligopeptidase activities. *Nat Prod Commun*. Nov 2013;8(11):1541-4.
3. Hulcova D, Marikova J, Korabecny J, et al. Amaryllidaceae alkaloids from N*arcissus pseudonarcissus* L. cv. Dutch Master as potential drugs in treatment of Alzheimer's disease. *Phytochemistry*. Sep 2019;165:112055. doi:10.1016/j.phytochem.2019.112055
4. Breiterová K, Koutová D, Maříková J, et al. Amaryllidaceae Alkaloids of Different Structural Types from Narcissus L. cv. Professor Einstein and Their Cytotoxic Activity. *Plants*. 2020;9(2):137.
5. Safratova M, Hostalkova A, Hulcova D, et al. Alkaloids from *Narcissus poeticus* cv. Pink Parasol of various structural types and their biological activity. *Arch Pharm Res*. Feb 2018;41(2):208-218. doi:10.1007/s12272-017-1000-4
6. Vaněčková N, Hošt'álková A, Šafratová M, et al. Isolation of Amaryllidaceae alkaloids from Nerine bowdenii W. Watson and their biological activities. 10.1039/C6RA20205E. *RSC Advances*. 2016;6(83):80114-80120. doi:10.1039/c6ra20205e
7. Hošťálková A, Novák Z, Hrabinová M, et al. Alkaloids from Berberis vulgaris and their biological activity connected to Alzheimer's disease. *Planta Medica*. 2015;81(16)doi:10.1055/s-0035-1565750

8. Opletal L, Locarek M, Frankova A, et al. Antimicrobial activity of extracts and isoquinoline alkaloids of selected papaveraceae plants. *Nat Prod Commun*. Dec 2014;9(12):1709-12.
9. Chlebek J, Macáková K, Cahlíková L, Kurfürst M, Kuneš J, Opletal L. Acetylcholinesterase and Butyrylcholinesterase Inhibitory Compounds from Corydalis Cava (Fumariaceae). *Natural Product Communications*. 2011;6(5). doi:10.1177/1934578x1100600507
10. Cipra G. *Biological activity of plants metabolites.XVII.Alkaloids of Corydalis yanhusuo W.T.WANG.* Diploma thesis. Univerzita Karlova, Farmaceutická Fakulta v Hradci Králové; 2011.
11. Cahlíková L, Macáková K, Kuneš J, et al. Acetylcholinesterase and Butyrylcholinesterase Inhibitory Compounds from Eschscholzia californica (Papaveraceae). *Natural Product Communications*. 2010;5(7)doi:10.1177/1934578x1000500710
12. Chlebek J, Novak Z, Kassemova D, et al. Isoquinoline Alkaloids from Fumaria officinalis L. and Their Biological Activities Related to Alzheimer's Disease. *Chem Biodivers*. Jan 2016;13(1):91-9. doi:10.1002/cbdv.201500033
13. Puzyrevská J. *Biological activity of plant metabolites XXXIV. Alkaloids from the herb of Glaucium flavum CRANTZ and their impact on human cholinesterases*. Diploma thesis. Univerzita Karlova, Farmaceutická Fakulta v Hradci Králové; 2017.
14. Cahlikova L, Opletal L, Kurfurst M, Macakova K, Kulhankova A, Hostalkova A. Acetylcholinesterase and butyrylcholinesterase inhibitory compounds from *Chelidonium majus* (Papaveraceae). *Nat Prod Commun*. Nov 2010;5(11):1751-4. doi:10.1177/1934578x1000501110
15. Rosiková T. *Biological activity of plant metabolites. XV. Alkaloids of Papaver somniferum L. and their activity to acetylcholinesterase*. Diploma thesis. Univerzita Karlova, Farmaceutická Fakulta v Hradci Králové; 2010.
16. Filáková I. *Biological activity of plant metabolites. XVI. Alkaloids of Papaver somniferum L. and their activity to acetylcholinesterase*. Diploma thesis. Univerzita Karlova, Farmaceutická Fakulta v Hradci Králové; 2010.
17. Suchánková P. *Biological activity of plant metabolites. XIV. Alkaloids of Papaver somniferum L. and their activity to acetylcholinesterase.* Univerzita Karlova, Farmaceutická Fakulta v Hradci Králové; 2010.
18. Host'alkova A, Opletal L, Kunes J, et al. Alkaloids from Peumus boldus and their acetylcholinesterase, butyrylcholinesterase and prolyl oligopeptidase inhibition activity. *Nat Prod Commun*. Apr 2015;10(4):577-80.
19. Klíčová A. *Alkaloids from wood of the species Liriodendron tulipifera L. and their biological activity*. Diploma. Univerzita Karlova, Farmaceutická Fakulta v Hradci Králové; 2018.
20. Hrušková M. *Alkaloids from the wood of the species Liriodendron tulipifera L. and their activity against human cholinesterases*. Diploma. Univerzita Karlova, Farmaceutická Fakulta v Hradci Králové; 2018.
21. Hsieh CF, Jheng JR, Lin GH, et al. Rosmarinic acid exhibits broad anti-enterovirus A71 activity by inhibiting the interaction between the five-fold axis of capsid VP1 and cognate sulfated receptors. *Emerg Microbes Infect*. Dec 2020;9(1):1194-1205. doi:10.1080/22221751.2020.1767512
22. Tang WF, Tsai HP, Chang YH, et al. Perilla (Perilla frutescens) leaf extract inhibits SARS-CoV-2 via direct virus inactivation. *Biomed J*. Jun 2021;44(3):293-303. doi:10.1016/j.bj.2021.01.005
23. Chen YL, Chen CY, Lai KH, Chang YC, Hwang TL. Anti-inflammatory and antiviral activities of flavone C-glycosides of Lophatherum gracile for COVID-19. *J Funct Foods*. Feb 2023;101:105407. doi:10.1016/j.jff.2023.105407
24. Morris GM, Huey R, Lindstrom W, et al. AutoDock4 and AutoDockTools4: Automated docking with selective receptor flexibility. *J Comput Chem*. Dec 2009;30(16):2785-91. doi:10.1002/jcc.21256
25. Korinek M, Tsai YH, El-Shazly M, et al. Anti-allergic Hydroxy Fatty Acids from Typhonium blumei Explored through ChemGPS-NP. *Front Pharmacol*. 2017;8:356. doi:10.3389/fphar.2017.00356
26. Topcu G, Şenol H, Alim Toraman GÖ, VM. A. Natural Alkaloids as Potential Anti-Coronavirus Compounds. *Bezmialem Science* 2020;8:131-139. doi:10.14235/bas.galenos.2020.5035

27. Zhang L, Lin D, Kusov Y, et al. alpha-Ketoamides as Broad-Spectrum Inhibitors of Coronavirus and Enterovirus Replication: Structure-Based Design, Synthesis, and Activity Assessment. *J Med Chem*. May 14 2020;63(9):4562-4578. doi:10.1021/acs.jmedchem.9b01828
28. Antonio ADS, Wiedemann LSM, Veiga-Junior VF. Natural products' role against COVID-19. *RSC Adv*. Jun 16 2020;10(39):23379-23393. doi:10.1039/d0ra03774e
